# *HOXD8* hypermethylation as a fully sensitive and specific biomarker for biliary tract cancer detectable in tissue and bile samples

**DOI:** 10.1038/s41416-022-01738-1

**Published:** 2022-02-17

**Authors:** Eleonora Loi, Cesare Zavattari, Alessandro Tommasi, Loredana Moi, Matteo Canale, Agnese Po, Claudia Sabato, Ana Florencia Vega-Benedetti, Pina Ziranu, Marco Puzzoni, Eleonora Lai, Luca Faloppi, María Rullán, Juan Carrascosa, Irene Amat, Jesús M. Urman, Maria Arechederra, Carmen Berasain, Elisabetta Ferretti, Andrea Casadei-Gardini, Matías A. Avila, Sergio Alonso, Mario Scartozzi, Patrizia Zavattari

**Affiliations:** 1 Department of Biomedical Sciences, Unit of Biology and Genetics, University of Cagliari, 09042 Cagliari, Italy; 2 Tennis Commander Srl, 56123 Pisa, Italy; 3 Biosciences Laboratory - IRCCS Istituto Romagnolo per lo Studio dei Tumori (IRST) “Dino Amadori”, 47014 Meldola, Italy; 4 Department of Molecular Medicine, Sapienza University of Rome, 00161 Rome, Italy; 5 Department of Experimental Medicine, Sapienza University of Rome, 00161 Rome, Italy; 6 Department of Medical Oncology, University Hospital of Cagliari, 09042 Cagliari, Italy; 7 Medical Oncology Unit, Macerata General Hospital, ASUR Marche AV3, 62100 Macerata, Italy; 8 Department of Gastroenterology and Hepatology, Navarra University Hospital Complex, 31008 Pamplona, Spain; 9 Instituto de Investigaciones Sanitarias de Navarra IdiSNA, 31008 Pamplona, Spain; 10 Department of Pathology, Navarra University Hospital Complex, 31008 Pamplona, Spain; 11 Hepatology Program, CIMA, University of Navarra, 31008 Pamplona, Spain; 12 CIBERehd, Instituto de Salud Carlos III, 28029 Madrid, Spain; 13 Vita-Salute San Raffaele University, 20132 Milan, Italy; 14 Program of Predictive and Personalized Medicine of Cancer, Germans Trias i Pujol Research Institute (PMPPC-IGTP), Badalona, Spain

**Keywords:** Diagnostic markers, Biliary tract cancer

## Abstract

**Background:**

Biliary tract cancers (BTC) are rare but highly aggressive tumours with poor prognosis, usually detected at advanced stages. Herein, we aimed at identifying BTC-specific DNA methylation alterations.

**Methods:**

Study design included statistical power and sample size estimation. A genome-wide methylation study of an explorative cohort (50 BTC and ten matched non-tumoral tissue samples) has been performed. BTC-specific altered CpG islands were validated in over 180 samples (174 BTCs and 13 non-tumoral controls). The final biomarkers, selected by a machine-learning approach, were validated in independent tissue (18 BTCs, 14 matched non-tumoral samples) and bile (24 BTCs, five non-tumoral samples) replication series, using droplet digital PCR.

**Results:**

We identified and successfully validated BTC-specific DNA methylation alterations in over 200 BTC samples. The two-biomarker panel, selected by an *in-house* algorithm, showed an AUC > 0.97. The best-performing biomarker (chr2:176993479-176995557), associated with *HOXD8*, a pivotal gene in cancer-related pathways, achieved 100% sensitivity and specificity in a new series of tissue and bile samples.

**Conclusions:**

We identified a novel fully efficient BTC biomarker, associated with *HOXD8* gene, detectable both in tissue and bile by a standardised assay ready-to-use in clinical trials also including samples from non-invasive matrices.

## Background

Biliary tract cancer (BTC) comprises a group of highly aggressive malignancies clinically classified as intrahepatic and extrahepatic cholangiocarcinomas (CCAs) and gallbladder cancers (GBCs).

BTC incidence and mortality vary according to geographic regions and are related to the distribution of risk factors associated with this cancer [1]. In Western countries, the main risk factors for CCA include biliary tract diseases such as benign stenosis, primary sclerosing cholangitis (PSC), hepatolithiasis and choledochal cysts, and these tumours display low but gradually increasing incidence rates [2]. Early detection of CCA in PSC patients is difficult since the associated-inflammatory process leads to biliary strictures mimicking early neoplastic changes [3].

Due to its silent evolution and clinical manifestations only at advanced stages, BTCs are usually diagnosed when the tumour is locally advanced or metastatic, thus unresectable.

The current diagnostic strategy for BTC includes a combination of clinical, radiological, biochemical and histological approaches [4]. Endoscopic retrograde cholangiopancreatography (ERCP) combined with biliary brush cytology and cyto-histological analysis of tumour tissue could be performed to confirm a suspected case of BTC [5].

Regrettably, current diagnostic methods have shown limited specificity and sensitivity [6, 7]. The use of biomarkers is a promising alternative for the detection of BTC and some of them have already been implemented in the clinic, i.e., the carcinoembryonic antigen and the carbohydrate antigen 19-9 (CA 19-9). However, elevated levels of these markers have also been found in benign conditions challenging their specificity [4]. As a consequence, accurate diagnosis may prove challenging, highlighting the need for a detection method for BTC.

DNA methylation alterations are early events during tumorigenesis and may be detected as early as in preneoplastic lesions in many types of tumours [8–12], including CCA [13–15] and even several years prior to tumour diagnosis [16].

Several DNA methylation-based biomarkers, with the specificity of 100% and sensitivity values ranging between 75 and 89%, have been proposed to detect BTC in tissue samples [17–19]. However, many of these studies focused on biomarkers that are frequently hypermethylated also in other types of cancers, often with a higher incidence than BTC.

Genome-wide methylation profiling represents a promising strategy for the discovery of new biomarkers specific to BTC. To our knowledge, very few studies have conducted a global methylation analysis on BTC samples [20, 21].

An important advantage for clinical implementation is that methylation alterations can also be detected in cell-free DNA (cfDNA) from different matrices such as blood, urine and stool [8, 22–25], greatly facilitating their implementation in the clinical setting.

Several studies demonstrated that DNA methylation alterations can be detected in bile [13, 26], biliary brush cytology specimens [27, 28], plasma [18] and serum samples [29] from patients with BTC, although not always representing BTC-specific biomarkers.

In order to select BTC methylation alterations also detectable in non-invasive matrices such as blood and faeces, it becomes of crucial importance to identify extremely specific biomarkers for BTC and not for other cancers, especially those of gastrointestinal origin.

The primary aim of this work was to identify BTC-specific DNA methylation biomarkers, with high sensitivity and specificity regardless of the tumour localisation. We performed a whole-genome methylation profiling of 50 BTC tissue samples from different localisation (intrahepatic, extrahepatic and gallbladder) and ten matched-normal tissue samples using Illumina EPIC^®^ arrays. Secondly, we aimed at assessing the performance of the best biomarker, associated with *HOXD8* gene, in bile samples from BTC patients, using droplet digital PCR (ddPCR), currently the most sensitive technology. Finally, we tested whether this biomarker was specific for BTC or could also be detected in subjects at high risk of developing BTC, such as patients with benign stenosis.

## Methods

### Samples and data collection

#### Tissue samples

##### Discovery cohort for whole-genome methylation assay

Fifty formalin-fixed paraffin-embedded (FFPE) tumour tissue samples and ten matched-normal controls from BTC patients (25 males and 25 females, mean age at diagnosis: 70.4 ± 10.9, 22 intrahepatic, 20 gallbladders and 8 extrahepatic) were obtained from the Oncology Service, Department of Medical Sciences and Public Health of the University of Cagliari (Italy) and IRCCS-Istituto Romagnolo per lo Studio dei Tumori (IRST) “Dino Amadori”, Meldola (Italy). Multiple haematoxylins and eosin slides were reviewed by an expert pathologist and sections including at least 50% of neoplastic tissue were selected as tumour samples, while sections devoid of malignant cells were selected as normal samples.

Samples were tested for CA 19-9 in the respective clinical centres.

Clinical characteristics of BTC patients are shown in Supplementary Table S1.

##### Validation cohort for droplet digital PCR assay

*Explorative tissue test series*: For an explorative analysis, 32 samples (14 paired BTC/normal samples, four BTC samples) were included. Nine out of 18 BTC were GBC and nine were CCA (6 extrahepatic, 3 intrahepatic). Five were overlapping with those analysed by Illumina EPIC arrays. FFPE samples were collected at Meldola centre mentioned above (Italy) (*N* = 28) and at the Department of Gastroenterology and Hepatology, Navarra University Hospital Complex, Pamplona (Spain) (*N* = 4).

Samples were tested for CA 19-9 in the respective clinical centres.

Clinical data are presented in Supplementary Table S2.

#### Publicly available datasets

Methylation data from The Cancer Genome Atlas (TCGA) and GEO portal dataset were retrieved for the validation of the identified methylation alterations. Additional details are provided in Supplementary Document S1.

#### Bile samples

Twenty-nine bile samples, comprising 24 samples from a new series of CCA patients (21 extrahepatic and 3 intrahepatic; four of which were paired with tissue samples), and five from patients with benign stenosis, were included. Bile samples were collected during ERCP at Pamplona (Spain) centre mentioned above as previously described [30]. After collection bile samples were maintained at 4 °C, centrifuged for 10 min (4 °C) at 3500 × *g* and stored in aliquots at −80 °C in a biobank facility. The whole process was performed in less than 2 h.

Samples were tested for CA 19-9.

Clinical data are reported in Supplementary Table S3.

### Experimental assays

#### DNA extraction and quantification

DNA was extracted from FFPE tissues using QIAamp DNA FFPE Tissue kit (Qiagen) or the QIAamp DNA kit (Qiagen). DNA was extracted by microdissection of five FFPE tissue slides of 10 µm and 20 µm. DNA concentration was quantified by UV spectrophotometry (NanoDrop Products, Thermo Scientific) and by fluorometric reading (Quant-iT™ PicoGreen^®^ dsDNA Assay Kit).

cfDNA was extracted from 1 ml of bile. Prior to cfDNA isolation, bile was thawed at 4 °C and centrifugated at 14,000 × *g* for 10 min at 4 °C to ensure the removal of impurities in the supernatant. Bile cfDNA was extracted using the Maxwell RSC ccfDNA Plasma Kit (Promega) according to the manufacturer’s instructions. Bile cfDNA concentrations were determined using a QuantiFluor dsDNA System (Promega), and cfDNA size distributions were analysed by Agilent 2100 Bioanalyzer (Agilent Technologies).

#### Genome-wide methylation assay

The quality of DNA extracted from FFPE samples was evaluated prior to bisulfite conversion using Infinium HD FFPE QC Assay (Illumina). DNA samples that passed this quality control step were treated with bisulfite using EZ DNA Methylation Gold Kit (Zymo Research). Bisulfite-converted DNA samples were subjected to a DNA restoration process using Infinium FFPE DNA Restore Kit (Illumina).

DNA samples were analysed by Illumina Infinium Human Methylation EPIC BeadChips (EPIC) interrogating over 850,000 CpG sites, according to the Illumina Infinium^®^ HD Methylation protocol. Illumina iScan was used to scan and record high-resolution images of the emitted fluorescence.

#### Droplet digital PCR assay

##### Bisulfite treatment

DNA samples were treated with bisulfite using EZ DNA Methylation Gold Kit (Zymo Research).

##### Droplet digital PCR DNA methylation assays

Primer and probes were designed for the two assays targeting CpG islands (CGIs) at chr2:176993479-176995557 and chr5:145713641-145713913.

The assays were designed on the genomic regions selected based on the methylation information of the CpG sites interrogated by the methylation array probes in the CGIs of interest. In particular, only regions including CpG sites displaying low methylation values in normal samples and high methylation values in tumour samples from the Discovery, TCGA-CHOL and GSE89803 datasets, were selected. DNA methylation status was analysed by ddPCR using the QX200^TM^ Droplet Digital^TM^ PCR System (BioRad) as previously described [31]. The ddPCR reaction comprised primers (900 nM each), probes (250 nM each), 30 ng bisulfite-converted DNA template from tissues and 70 ng bisulfite-converted DNA template from bile and 1× ddPCR Supermix for Probes (BioRad) in a final volume of 22 µL. The 4Plex control was included in all wells (for sequences for the 4Plex control see ref. [31]). Droplets were generated in the QX200 Droplet Generator (BioRad), with 70 µL of droplet generation oil (BioRad) and 20 µL ddPCR mixture and the PCR was carried out in a T100 Thermal Cycler (BioRad) using the cycling programme recommended by the manufacturer. Finally, the QX200 Droplet Reader (BioRad) was used to read the fluorescence signals.

### Data analyses

The detailed analysis of genome-wide and ddPCR methylation data is described in Supplementary Document S1.

For the selection of the most informative biomarkers, an in-house algorithm named TASTOPAL (The Accurate System TO Predict A Lump) was developed. Additional details and the selection pipeline are available in Supplementary Document S1. Supplementary Fig. S4 shows an example of methods applied by the algorithm to select the best biomarkers.

### Statistical analyses and power calculation

Chi-square and Fisher’s exact tests (two-sided) were used to determine statistically significant associations between DNA methylation patterns and *vs* clinical parameters. Statistical analyses of methylation data and power calculation are described in the relative data analysis paragraphs in Supplementary Document S1.

## Results

The experimental strategy to identify highly specific and sensitive BTC methylation-based biomarkers consisted of: (1) global DNA methylation analysis of the Discovery cohort samples; (2) validation of the results in TCGA-CHOL cohort and GSE89803 cohort samples; (3) selection of methylation alterations not shared with other gastrointestinal tumours, particularly those with high incidence, i.e. colon cancer (TCGA-COAD), rectal cancer (TCGA-READ) and gastric cancer (TCGA-STAD); (4) application of a machine-learning approach to select the minimum number of the most specific and sensitive alterations; (5) identification of the best-performing biomarker, tested in bile samples.

**Fig. 1:**
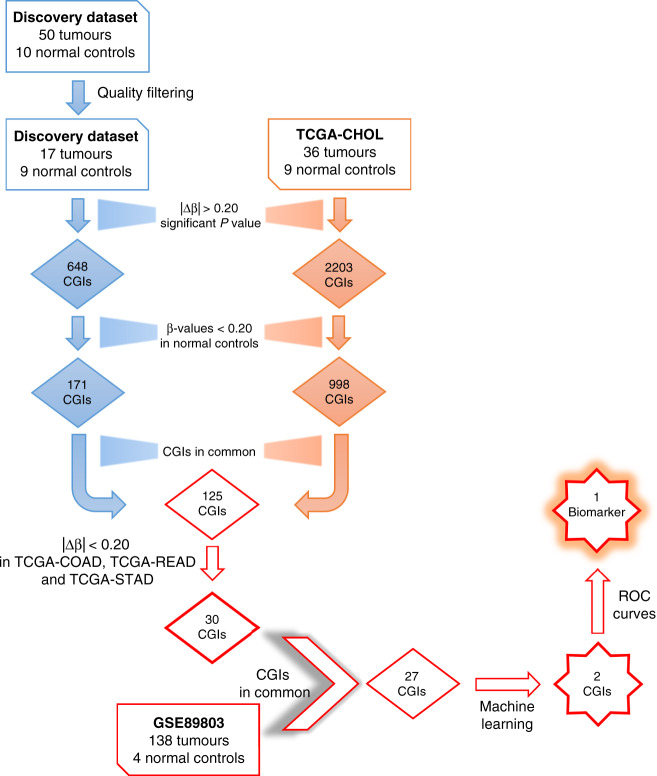
Analysis workflow. Workflow of DNA methylation alterations selection: from a genome-wide to a targeted CpG island approach.

Figure 1 summarises the analysis workflow.

### Identification of DNA methylation alterations in the Discovery dataset

A genome-wide methylation analysis of 50 tumours and ten matched-normal tissue samples from 50 BTC patients was performed using Illumina EPIC arrays. According to sample size calculation (see Supplementary Document S1), the analysed number of samples would guarantee to identify biologically relevant differences in methylation (|Δβ | = 0.2) with a statistical power of 100%. After filtering samples based on the β-values distribution (Supplementary Fig. S1), 26 good-quality samples, comprising 17 tumours and nine matched-normal samples, were selected for the downstream analyses.

**Fig. 2:**
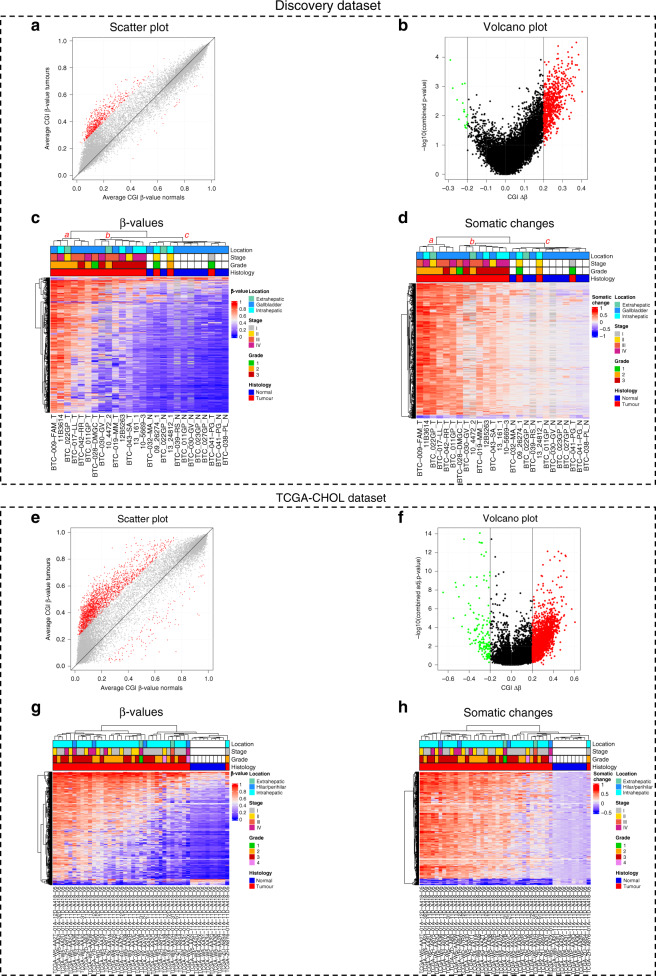
Visualisation of genome-wide analysis results in the Discovery and TCGA-CHOL datasets. **a** Scatter plot displaying average CGI β-values distribution in normal and tumour samples of the Discovery dataset. Red dots indicate statistically significant differentially methylated (|Δβ| > 0.20, *P* value < 0.05) CGIs. **b** Volcano plot of CGIs Δβ of the Discovery dataset. In the *x* axis, the difference of methylation between tumours and normal samples. In the *y* axis, the -logarithm of the *P* value. Red dots indicate hypermethylated CGIs (Δβ > 0.2) and green dots represent hypomethylated CGIs (Δβ < −0.2). **c**, **d** Discovery dataset unsupervised hierarchical clustering analysis based on the average CGI β values (**c**) and on the average CGI somatic changes, defined as the difference between the β-value of every tumour and the average of the normal samples for each of the aberrantly methylated CGIs (**d**). **e** Scatter plot displaying average CGI β-values distribution in normal and tumour samples of the TCGA-CHOL dataset. Red dots indicate statistically significant differentially methylated CGIs. **f** Volcano plot distribution of CGIs Δβ of the TCGA-CHOL dataset. Red dots indicate hypermethylated CGIs (Δβ > 0.2) and green dots represent hypomethylated CGIs (Δβ < −0.2). **g**, **h** TCGA-CHOL dataset unsupervised hierarchical clustering analysis based on the average CGI β values (**g**) and somatic changes (**h**) for each of the aberrantly methylated CGIs.

The differential methylation analysis between tumour and matched-normal samples identified 648 differentially methylated (|Δβ| > 0.20, combined *P* value < 0.05) CpG islands (CGIs), comprising 631 hypermethylated (Δβ > 0.20) and 17 hypomethylated (Δβ < −0.20) CGIs (Fig. 2a, b). No statistically significant differentially methylated CGI was detected after *P* value correction for multi-hypothesis testing (using FDR). This was not entirely unexpected given the reduced sample size in the analysis [8, 16, 32, 33].

Two heatmaps were generated using CGI methylation values (Fig. 2c, d). Unsupervised hierarchical clustering analysis (UHC) yielded two main clusters: a small cluster comprising the most hypermethylated tumour samples (cluster *a*) and a second cluster further divided into a cluster of tumours showing intermediate methylation values (cluster *b*) and a cluster including the normal samples along with three tumour samples showing low methylation values (cluster *c*) (Fig. 2c, d). No statistically significant association was observed between clusters and tumour location. Stage I and II tumours predominantly clustered with normal controls (cluster c) compared with tumours of higher Stages (III and IV) that were in the cluster including only tumours (clusters a and b) (Fisher’s test *P* value = 0.015). Grade 2 tumours were predominantly in cluster *a*, while grade 3 tumours were in cluster *b* (Fisher’s test *P* value = 0.029).

We focused on CGIs that become hypermethylated in tumours, under the rationale that they would be more easily detected in liquid biopsies than CGIs that become hypomethylated. Moreover, CGIs showing β-values higher than 0.20 in normal samples (likely reflecting methylation heterogeneity among non-tumour cells) were filtered out, obtaining a final set of 171 somatically hypermethylated CGIs.

### Methylation analysis using TCGA-CHOL dataset

To increase the robustness of the identified methylation alterations, methylation data from TCGA-CHOL dataset (including 36 tumour samples and nine normal tissue samples) were analysed. Differential methylation analysis revealed 2203 differentially methylated CGIs (|Δβ| > 0.20, adjusted combined *P* value < 0.05) (Fig. 2e, f). This dataset yielded a higher number of both hypermethylated (2078) and hypomethylated (125) CGIs compared to the Discovery dataset.

UHC of the TCGA-CHOL samples was similar to that observed in the Discovery dataset, with only one tumour sample clustering in the normal sample subgroup (Fig. 2g, h). No significant associations with tumour stage or grade were observed.

Again, we focused on hypermethylated CGIs in tumours. CGIs showing β-values higher than 0.20 in normal samples were filtered out resulting in 998 hypermethylated CGIs. Notably, 125 out of 171 hypermethylated CGIs (*P* value = 1.5 × 10^−9^) identified in our Discovery dataset were validated in the TCGA-CHOL dataset (Fig. 1).

### Selection of BTC-specific methylation alterations

**Fig. 3:**
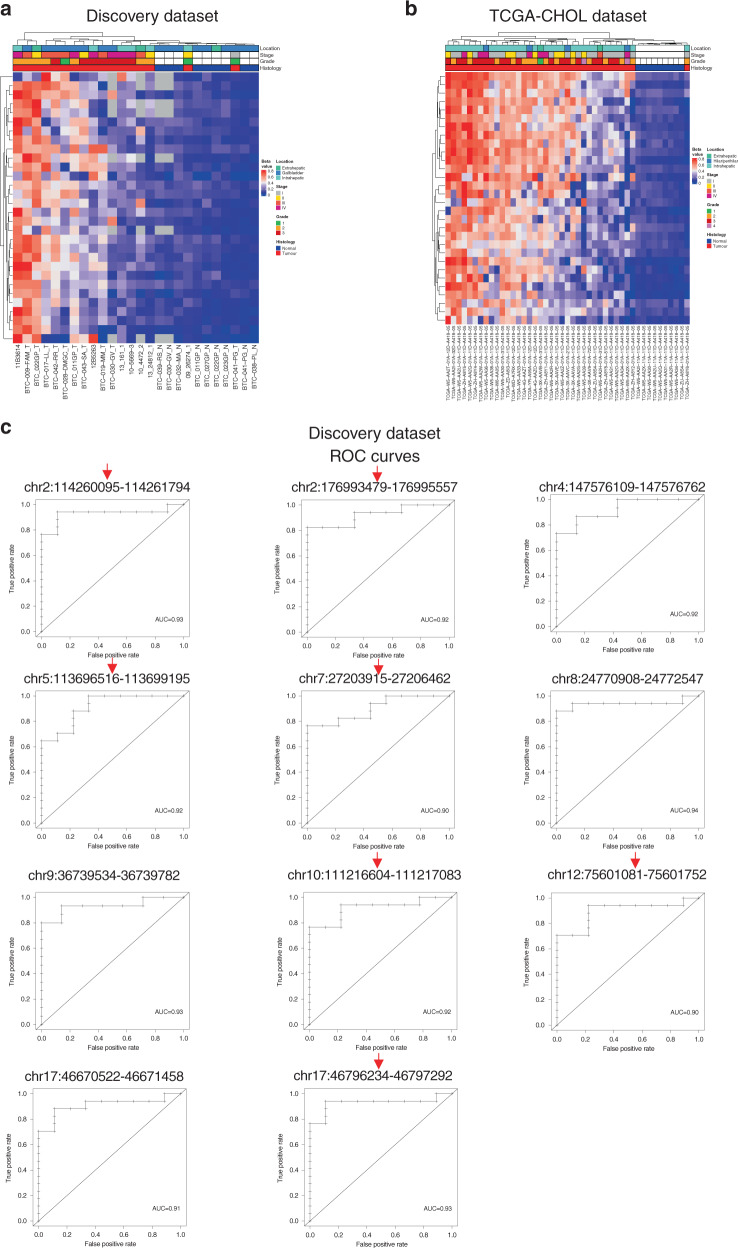
Visualisation of 30 selected altered CpG islands analysis in the Discovery and TCGA-CHOL datasets. **a**, **b** Discovery dataset (**a**) and TCGA-CHOL dataset (**b**) unsupervised hierarchical clustering analysis based on the average CGI β-values for the 30 BTC-specific altered CGIs. **c** ROC curves for the 11 CGIs showing an AUC ≥ 0.90 in the Discovery dataset. Red arrows indicate CGIs showing an AUC ≥ 0.90 also in TCGA-CHOL dataset.

To select only BTC-specific alterations, we analysed the methylation changes of the putative biomarkers in other gastrointestinal cancer types using data from TCGA. Of the 125 previously validated CGIs, we excluded those that also exhibited differential methylation (|Δβ| > 0.20) in any of the colon (COAD), rectal (READ) or gastric (STAD) cancer datasets of the TCGA, obtaining a set of 30 BTC-specific somatically hypermethylated CGIs (Supplementary Table S4). Heatmaps generated with methylation values of these CGIs in the Discovery (Fig. 3a) and TCGA-CHOL (Fig. 3b) datasets showed very similar clustering (Fig. 2c, g).

Specificity and sensitivity of the 30 BTC-specific methylation-based biomarkers were evaluated by ROC analysis. In the Discovery dataset, 11 CGIs showed an area under curve (AUC) equal or higher than 0.90 (Fig. 3c), while 21 CGIs had an AUC ≥ 0.90 in TCGA-CHOL dataset. Seven CGIs had an AUC equal or higher than 0.90 in both datasets (Supplementary Table S5).

### BTC-specific altered CGIs in the excluded samples

**Fig. 4:**
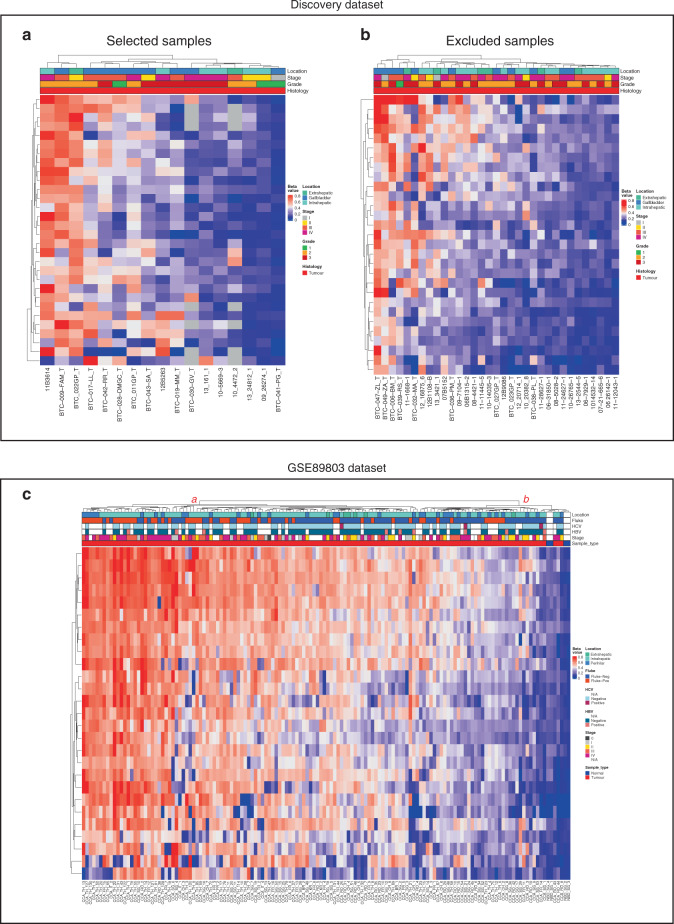
Heatmaps showing the replication results of the selected altered CpG islands in the Discovery and GSE89803 datasets. **a**, **b** Discovery dataset good-quality selected samples (**a**) and excluded samples (**b**) unsupervised hierarchical clustering analysis based on the average CGI β-values for the 30 BTC-specific altered CGIs. **c** GSE89803 dataset unsupervised hierarchical clustering analysis based on the average CGI β-values for the 27 validated CGIs.

To explore the behaviour of the selected methylation alterations in the 33 tumour samples that were initially excluded due to their abnormal β-value distributions (Supplementary Fig. S1), UHC was carried out using CGI methylation values of the 30 BTC-specific altered CGIs. The rationale of this analysis was that despite the abnormal genome-wide β-value distribution of these samples, the β-values exclusive of the selected CGIs could still provide useful information. The results revealed that the clustering of these samples (Fig. 4b) was similar to that of the good-quality selected tumour samples (Fig. 4a), reinforcing the potential value of these alterations for BTC detection.

### Validation in a large BTC dataset

In order to validate alterations of the 30 selected CGIs, methylation data of a large dataset (GSE89803) including 138 tumours and four normal tissue controls from different ethnic groups were analysed. Methylation alterations (|Δβ| > 0.20) were confirmed for 27 out of 30 CGIs. Of note, two of the three CGIs that were not validated displayed high methylation values in both tumour and normal samples (Supplementary Table S6).

UHC of GSE89803 samples yielded similar results to those observed for the Discovery and TCGA-CHOL datasets, with only five tumour samples clustering along with the normal ones (Fig. 4c).

### Selection of best-performing biomarkers

**Fig. 5:**
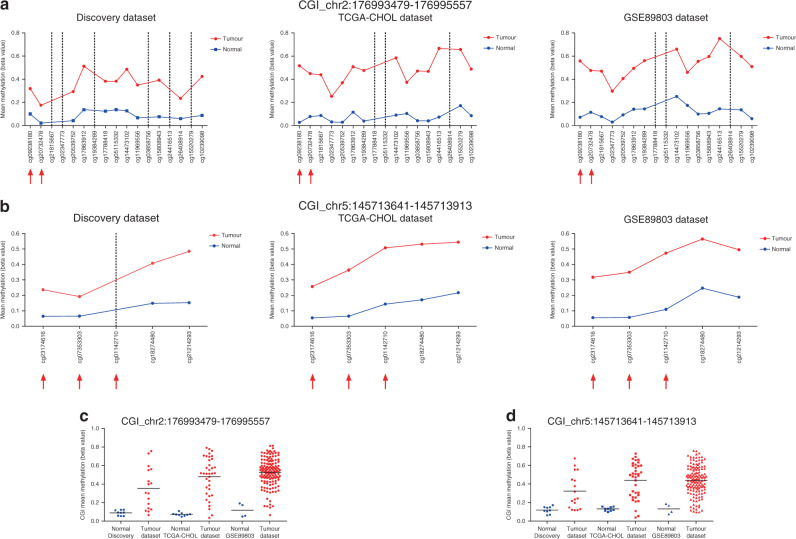
Methylation values of the CpG sites within the two CpG islands selected by a machine-learning approach in the Discovery, TCGA-CHOL and GSE89803 datasets. **a**, **b** Methylation values obtained from the Discovery (EPIC array), TCGA-CHOL (450 K array) and GSE89803 (450 K array) datasets. Mean β-values, resulting from the average of the samples (normal indicated with blue dots and tumours indicated with red dots), of each probe, belonging to CGI chr2:176993479-176995557 (**a**) and CGI chr5:145713641-145713913 (**b**). The red arrows indicate the CpG sites included in ddPCR experimental assay design. **c**, **d** Box plots of the CGI mean β-values for CGI chr2:176993479-176995557 (**c**) and CGI chr5:145713641-145713913 (**d**) in tumour and normal tissues obtained from the Discovery, TCGA-CHOL and GSE89803 datasets.

The application of a Machine-Learning approach, using the Discovery dataset as training set to prioritise methylation biomarkers and build a diagnostic model on them, and TCGA-CHOL and GSE89803 as validation datasets, resulted in an extremely compact model which generated a ranking list of the best combinations of biomarkers (based on AUC, sensitivity and specificity). We selected the best combination in terms of very high performance and technical assay feasibility. The two biomarkers combination (CGIs mapping on chr2:176993479-176995557 and chr5:145713641-145713913), achieved a promising AUC = 0.972, sensitivity = 0.944 and specificity=1.00 on the TCGA dataset, and AUC = 0.982, sensitivity = 0.964 and specificity = 1.00, on the GSE89803 dataset. Figure 5 shows methylation values of the CpG sites interrogated by EPIC probes in the selected CGIs across the three different datasets.

### Validation of best-performing biomarkers

As a further step towards the future implementation of these biomarkers in clinical settings, we explored the application of digital PCR DNA methylation assays on ten (five matched tumour and normal) samples previously profiled by Illumina EPIC arrays and 69 additional tissue and bile samples from new case series.

According to sample size calculation (see Supplementary Document S1), the estimated statistical power to identify biologically relevant differences in methylation (|Δβ | = 0.2) was 100% for the tissue cohort and 99.97% for the bile cohort. Removing the 10 tissue samples previously analysed by EPIC arrays, thus reducing the sample size to 13 BTC and 9 normal tissues, the estimated statistical power remained very high (99.996%).

#### Exploratory analyses—tissue samples

**Fig. 6:**
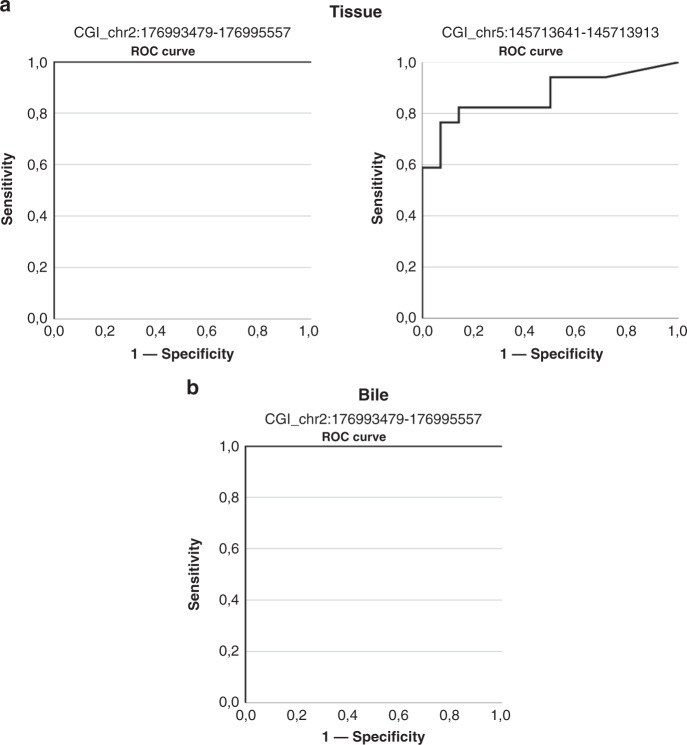
ROC curves of the two CpG islands selected by a machine-learning approach in tissues and liquid biopsies. **a** ROC curves relative to CGI chr2:176993479-176995557 (on the left) and CGI chr5:145713641-145713913 (on the right) biomarkers in tissue samples. **b** ROC curve relative to CGI chr2:176993479-176995557 biomarker in bile samples.

The two candidate biomarkers were tested in a series of BTC (*N* = 18) and paired normal (*N* = 14) tissue samples. Assay chr2:176993479-176995557 showed a sensitivity of 100% (*N* = 17/17), a specificity of 100% (*N* = 14/14) and AUC of 1.00 (Fig. 6a), while the sensitivity of assay chr5:145713641-145713913 was 76% (*N* = 13/17), the specificity was 93% (*N* = 13/14) and AUC was 0.870 (Fig. 6a). One tumour tissue sample was excluded since it resulted negative for both biomarkers. Of note, this sample was already excluded in the whole-genome methylation analysis because of its abnormal β-value distribution (Supplementary Fig. S2). The combined two-biomarker panel resulted in 100% (*N* = 17/17) sensitivity and 93% (*N* = 13/14) specificity.

#### Exploratory analyses—bile samples

Since assay chr2:176993479-176995557 exhibited the highest sensitivity and specificity in tissue samples, bile samples were tested using only this best biomarker. Bile series comprised 24 samples from BTC patients and five samples from patients with benign biliary stenosis. Despite the reduced number of non-tumoral controls, both the sensitivity and the specificity were 100% and the AUC was 1.00 (Fig. 6b).

## Discussion

BTCs are extremely aggressive tumours that metastasise in most of patients. It is therefore urgent to identify stable and easily traceable biomarkers ideally available by non-invasive or minimally invasive monitoring approaches. DNA methylation alterations respond to this need, representing specific tumour signatures and much more stable than RNA and proteins.

Moreover, DNA methylation alterations are very early events in carcinogenesis, thus representing extremely valuable biomarkers not only as sentinels of relapse, minimal residual disease or metastasis but also for primary diagnosis. BTCs are indeed diagnosed at an advanced stage due to the lack of accurate diagnostic methods.

While the identification of tumour location-specific methylation profiles may be important [34–36], in this study we identified DNA methylation alterations that could enable detection and tracking of biliary tract tumours regardless of their anatomical location and natural history. This suggests that the methylation profile of these tumours might present a lowest common denominator among different pathological subtypes.

The whole-genome methylation approach allowed us to identify and validate 27 BTC-specific methylation alterations on three large case series (Discovery, TCGA-CHOL and GSE89803 datasets).

Several DNA methylation biomarkers have been proposed to detect BTC. These biomarkers, however, are also frequently hypermethylated in other more prevalent gastrointestinal cancers [17, 18, 28]. To increase the specificity of our putative biomarkers, we selected CGIs not altered in other gastrointestinal tumours (colon, rectal and gastric cancer). The identification of BTC-specific alterations is crucial when investigated in non-invasive matrices, such as blood and stool samples, but also in minimally invasive matrices such as bile, to avoid misclassification with other malignancies.

A proprietary machine-learning algorithm (TASTOPAL) was developed to select the most informative CGIs among the altered ones. This approach proved successful because it rendered a panel of only two markers showing very high sensitivity and specificity in three independent datasets of BTC patients (from different ethnic groups, tumour locations, predisposing risk factors). Importantly, the same algorithm can be applied for the selection of markers of other tumour types and different diseases, with manifold advantages compared to the manual biomarker selection.

Illumina Methylation EPIC arrays have proven to be an excellent tool to discover novel potential biomarkers. We have assessed the translatability of the DNA methylation results obtained by EPIC arrays towards droplet digital PCR (ddPCR), an extremely sensitive, robust, fast and cost-effective technique, ideal for the absolute quantification of low-copies DNA molecules of interest.

The two-biomarker panel showed a very high performance in tissue samples (combined sensitivity of 100% and specificity of 93%). A perfect concordance between the results obtained for the same five paired tissue samples by the genome-wide and the targeted methylation assays, specifically designed for a selected region within the two altered CGIs, was observed (data not shown). Of note, BTC samples were equally represented by bile ducts (*N* = 9) and gallbladder (*N* = 9) tumours reinforcing the potential of this biomarker panel to detect tumours from different localisations.

The performance of the two individual ddPCR assays was not identical, possibly due to the assay design. The chr2:176993479-176995557 was more accurate (100% sensitivity and specificity) in tissue samples. For this reason, it was selected as the best biomarker to be tested in bile samples to verify its performance as a minimally invasive detection tool for BTC. Of note, the biomarker has shown excellent (>0.90) AUC values in all the datasets analysed. Interestingly, a positive correlation between the statistical power and the robustness of the biomarker was observed (Supplementary Fig. S3).

The biomarker showed 100% sensitivity and specificity in bile samples, proving to be an excellent candidate biomarker for non-invasive BTC investigation. Moreover, this biomarker yielded negative results in bile samples from patients with benign biliary disease, suggesting that this methylation alteration is specific of a state of malignancy. Interestingly, one sample from a patient with benign stenosis showed three positive droplets for the marker of interest and using a threshold based on the best accuracy (another parameter that can be used for setting the threshold), rather than the highest sum of sensitivity and specificity, it was classified as positive. This would suggest that benign biliary disease patients resulting positive for this alteration may be at higher risk of developing BTC compared with negative ones and thus deserve a closer clinical follow-up. Moreover, the screening of this biomarker in patients with benign biliary diseases would be recommended. In fact, the presence of DNA methylation alterations in these patients may potentially represent early triggers in the carcinogenesis process and/or that they harbour pre-cancerous-like features many years prior to tumour onset. The development of BTC after 10–20 years of a previous benign pathology is not rare [37]. This possible scenario is also in line with the value of DNA methylation biomarkers as predictive of neoplasia, managing to forecast the development of cancer even ten years before onset [16].

According to our results, *HOXD8* hypermethylation is the best-performing biomarker to identify the presence of BTC regardless of tumour location. In fact, although previous works have demonstrated that DNA methylation alterations can be detected in bile and biliary brush samples, the proposed panels included a higher number of biomarkers and yet they showed lower sensitivity values. Moreover, the majority of these studies were focused on CCA or even on a particular CCA subtype, limiting the application of those panels for the detection of all BTC subtypes. For instance, Shin et al. proposed a five-biomarker panel, with a sensitivity of 76% and a specificity of 100%, to detect extrahepatic CCA in bile using MethyLight [26]. Another work found that *p14ARF* and *p16INK4a* methylation had respectively a sensitivity of 46% and 53% for the detection of CCA and GBC in bile [13]. However, similar or even higher percentages of PSC samples showed methylation of the two genes [13]. A six-gene panel allowed the distinction of malignant biliary strictures from benign samples with 77% sensitivity and 78% specificity [38]. Moreover, two studies analysing biliary brush samples, identified a three-biomarker panel, with specificity and sensitivity respectively of 86% and 80% [27], and a four-biomarker panel with 85% sensitivity and 98% specificity [28], distinguishing BTC patients from patients with other biliary diseases. Importantly, all the above-mentioned studies employed techniques with lower sensitivity compared to ddPCR [39].

Interestingly, the selected altered CGI (chr2:176993479-176995557) is associated with *HOXD8* gene, belonging to class I homeobox gene family, well known to be involved in carcinogenesis. This suggests that the selected alteration would fulfil the requirements of an optimal biomarker being the most informative but also very likely functionally relevant for the disease. Several studies have shown that *HOX* genes are either overexpressed or downregulated in a variety of cancers, acting respectively as proto-oncogenes or tumour suppressors depending on the tissue type. Epigenetic mechanisms, including DNA methylation and histone modifications, have been shown to be responsible for an altered expression of these genes in cancer. Specifically, *HOXD8* is epigenetically downregulated in lung cancer [40] and used as a biomarker to detect prostate cancer in urine samples [41]. Interestingly, *homeobox* genes are among the most hypermethylated genes in BTC [42] and, as reported above, other studies have identified other *HOX* genes as DNA methylation-based biomarkers for BTC, although with lower performances [29, 43].

We acknowledge some limitations in our study. First of all, the Discovery cohort comprised FFPE samples. This kind of sample represents a precious source for research purposes in those cases where it is extremely difficult to obtain fresh tumour samples. In the case of a rare tumour such as BTC, the availability of FFPE samples is even of greater value since the collection of samples for a study of adequate statistical power requires extraordinary time and efforts. However, formaldehyde induces several types of DNA damage such as crosslinks, DNA fragmentation, abasic sites and deamination of cytosine bases [44]. Importantly, formaldehyde-induced crosslinks, inter-strand DNA crosslinks and protein-DNA crosslinks may affect the efficacy of bisulfite treatment, by hampering DNA denaturation, crucial for strand-specific bisulfite conversion [45]. To overcome this limitation, it is imperative to restore DNA integrity after bisulfite treatment as performed in this study. Although this strategy has been successful to obtain reliable data, we cannot exclude that formalin fixation negatively affected the quality of the results from samples. In fact, we observed abnormal β-value distributions in some samples that were consequently removed from the analysis. Due to this selection process, the size of the Discovery cohort was substantially reduced, preventing the identification of methylation alterations resisting multiple-testing correction. We overcame this limitation by selecting DNA methylation alterations shared between the Discovery dataset and TCGA-CHOL dataset. Therefore, the robustness of our results is confirmed by the validation of the selected alterations in multiple independent datasets. Moreover, we showed that even the tumour samples initially excluded clustered similarly to the good-quality selected samples, when the methylation information only from the selected CGIs was used, further reinforcing the validity of our findings.

Another limitation of this study is the unavailability of tissues and bile samples from healthy individuals, for obvious technical and ethical reasons. Thus, the normal tissue samples derived from a section of the paraffin block devoid of malignant cells. Normal tissue surrounding the tumour could already show cancer characteristic alterations [46]. However, the normal samples of the analysed datasets displayed methylation profiles distinct from tumour samples and very similar among them as found in the distribution of the standard deviation (SD) of CpG methylation probes from the TCGA-CHOL dataset (median SD = 0.02, 90th percentile = 0.09). Of note, statistical power calculation has shown that the discovery cohort including 50 tumours and only ten matched-normal controls would guarantee 100% power to detect methylation alterations (|Δβ | ≥ 0.2) conventionally considered as biologically relevant (Supplementary Document S1).

Finally, we acknowledge that ddPCR results on bile samples would benefit from further validation in a large case series from different tumour locations and controls. Nevertheless, the number of analysed bile samples would guarantee a statistical power of 99.97% for the validation of the identified alterations (Supplementary Document S1). In agreement with the pathway to bring a candidate DNA methylation biomarker from the laboratory into molecular diagnostics [47], we have successfully completed the preclinical phase, including the definition of the biomarker, its external validation (in publicly available datasets), the assay design and external validation in independent cohorts (including both tissue and bile samples). Therefore, the promising results obtained during all these steps indicate that the biomarker is ready to be tested in a clinical trial.

In summary, we present a novel DNA methylation-based BTC biomarker, CGI chr2:176993479-176995557, associated with *HOXD8* gene, with excellent diagnostic capabilities, which can be applied both in tissue biopsies and bile samples, outperforming all previously reported biomarkers. The next challenge is to test this biomarker for the detection of BTC from completely non-invasive matrices, such as stools and blood. We also envision to begin a clinical trial to evaluate the impact of this biomarker in surveillance and early diagnostic tests of patients at risk for BTC development and to predict patient prognosis and response to treatments, improving patient stratification and personalised therapeutic strategies.

## Supplementary information

Reproducibility checklist

Supplementary Information

## Data Availability

The data that support the findings of this study are available from the corresponding author upon reasonable request.

## Code availability

The code to reproduce the results of this paper is available under reasonable request to the corresponding authors.
