# Supplementary material for: HOXD8 hypermethylation as a fully sensitive and specific biomarker for biliary tract cancer detectable in tissue and bile samples
Source: Br J Cancer. 2022 Feb 17;126(12):1783–94. doi: 10.1038/s41416-022-01738-1 (PMC9174245; doi:10.1038/s41416-022-01738-1)
Supplement: Supplementary file 2 — Supplementary Information [file 41416_2022_1738_MOESM2_ESM.docx]

**Supplementary data**

**Supplementary Tables**

Table S1

Table S2

Table S3

Table S4

Table S5

Table S6

Table S7

Table S8

**Supplementary Figures**

Fig. S1

Fig. S2

Fig. S3

Fig. S4

Fig. S5

Fig. S6

**Supplementary Material**

Supplementary Document S1

**Supplementary Tables**

**Table S1. Clinical characteristics of BTC patients of the Discovery cohort**

| **Patient ID** | **Gender** | **Age at tumour diagnosis** | **Tumour location** | **Grade** | **Stage at diagnosis** | **CA 19-9 (UI/ml)** | **Other clinical and pathological factors** |
| --- | --- | --- | --- | --- | --- | --- | --- |
| 06 26142-1 | M | 71 | Intrahepatic | 2 | II | 14 | / |
| 06-31850-1 | F | 77 | Intrahepatic | 2 | IIIA | 83 | / |
| 06-7929-1 | M | 66 | Intrahepatic | 3 | IIIC | 55 | / |
| 06B1315-2 | F | 44 | Intrahepatic | 2 | IV | 12 | / |
| 07-21-665-6 | M | 67 | Intrahepatic | 3 | IIIA | 34 | / |
| 07B5152 | F | 55 | Intrahepatic | 2 | IV | 325 | / |
| 08-4421-1 | M | 76 | Intrahepatic | 3 | IIIA | 17 | / |
| 08-5028-2 | F | 65 | Intrahepatic | 3 | IIIA | NA | / |
| 09_26274_1 | M | 77 | Intrahepatic | 1 | II | 33 | / |
| 09-7104-1 | M | 77 | Intrahepatic | 3 | IV | 547 | / |
| 10_20382_8 | F | 50 | Intrahepatic | 3 | II | 49 | / |
| 10_4472_2 | M | 66 | Extrahepatic | 2 | IIIA | 16 | Jaundice |
| 10-14035-3 | F | 68 | Extrahepatic | 2 | IIIC | 183 | Jaundice |
| 10-26765-1 | M | 58 | Gallbladder | 2 | IVB | 81 | / |
| 10-5669-3 | M | 70 | Intrahepatic | 3 | IV | 29 | / |
| 1014532-14 | M | 58 | Intrahepatic | 2 | IIIB | 9 | / |
| 11-11445-5 | M | 70 | Extrahepatic | 2 | IVA | 112 | Jaundice |
| 11-12043-1 | M | 65 | Intrahepatic | 3 | IV | NA | / |
| 11-1668-1 | M | 63 | Extrahepatic | 3 | IVB | 92 | Jaundice |
| 11-24627-1 | F | 60 | Gallbladder | 3 | VIB | 136 | / |
| 11-28627-1 | M | 67 | Extrahepatic | 3 | IVB | 826 | Jaundice |
| 11B3614 | F | 79 | Intrahepatic | 2 | IV | 1021 | / |
| 12_16875_6 | F | 64 | Intrahepatic | 2 | IIIA | 2284 | / |
| 12_20714_1 | M | 70 | Intrahepatic | 3 | IV | 14 | / |
| 12B1108-B | F | 66 | Intrahepatic | 2 | II | 123 | / |
| 12B5263 | M | 67 | Intrahepatic | 3 | IV | 31 | / |
| 12B9086 | F | 71 | Intrahepatic | 2 | IV | 61 | / |
| 13_161_1 | M | 90 | Intrahepatic | 3 | IV | 161 | / |
| 13_24812_1 | M | 61 | Intrahepatic | 2 | II | 39 | / |
| 13_3421_1 | M | 66 | Extrahepatic | 3 | I | 46 | / |
| 13-2544-5 | F | 65 | Extrahepatic | 2 | IIIC | 32 | / |
| BTC-006-BM | F | 79 | Gallbladder | 3 | IIIA | 651.5 | / |
| BTC-009-FAM | F | 84 | Gallbladder | 2 | IIIA | 1.8 | / |
| BTC-011-GP* | F | 71 | Gallbladder | 2 | IVB | 2197 | Gallbladder stones |
| BTC-017-LL | F | 70 | Gallbladder | 2 | IIIA | 78.1 | / |
| BTC-019-MM | F | 92 | Gallbladder | 3 | IIIA | 37.2 | / |
| BTC-022-PE* | M | 72 | Extrahepatic | 2 | II | 5.7 | / |
| BTC-023-PG*, # | F | 55 | Gallbladder | 2 | IIIB | 1.3 | / |
| BTC-027-BR* | F | 56 | Gallbladder | 2 | IVA | 3000 | / |
| BTC-028-DMGC | F | 57 | Gallbladder | 1 | IIIA | 35 | / |
| BTC-030-GV*, # | F | 82 | Gallbladder | 3 | IVA | 95.1 | / |
| BTC-032-MA*, # | M | 75 | Gallbladder | 3 | II | 10.5 | Gallbladder stones |
| BTC-036-PM*, # | F | 89 | Gallbladder | 2 | IIIA | 153.6 | / |
| BTC-038-PL*, # | F | 86 | Gallbladder | 2 | IVA | 234.7 | / |
| BTC-039-RS* | F | 87 | Gallbladder | 1 | IIIA | 1518 | / |
| BTC-041-PG* | M | 66 | Gallbladder | 1 | I | NA | Gallbladder stones |
| BTC-042-RR | M | 85 | Gallbladder | 3 | IIIB | 14.4 | / |
| BTC-043-SA | F | 78 | Gallbladder | 3 | II | 52.4 | / |
| BTC-047-ZL | M | 86 | Gallbladder | 3 | IVB | 15.9 | Gallbladder stones |
| BTC-049-ZA | M | 81 | Gallbladder | 2 | I | NA | / |

Notes: *including also normal-matched samples; NA: not available; #indicate samples also analysed by ddPCR

**Table S2. Clinical characteristics of BTC patients providing tissues for ddPCR**

| **Patient ID** | **Collection Center** | **Gender** | **Age at tumour diagnosis** | **Tumour location** | **Grade** | **Stage at diagnosis** | **CA 19-9 (UI/ml)** | **Other clinical and pathological factors** |
| --- | --- | --- | --- | --- | --- | --- | --- | --- |
| BTC-002-AA* | Italy | F | 77 | Gallbladder | 2 | IVB | 318.2 | / |
| BTC-012-GMP* | Italy | F | 83 | Gallbladder | 3 | IIIA | NA | gallbladder stones |
| BTC-013-GG* | Italy | M | 77 | Extrahepatic | 2 | IIIB | NA | / |
| BTC-014-GL* | Italy | M | 75 | Extrahepatic | 2 | IIIB | NA | / |
| BTC-035-PL* | Italy | M | 65 | Intrahepatic | 3 | IIIB | 639 | / |
| BTC-037-PD* | Italy | F | 72 | Gallbladder | 3 | IIIB | 47.9 | / |
| BTC-045-TL* | Italy | F | 84 | Gallbladder | 2 | II | 195.3 | / |
| BTC-053-FA* | Italy | M | 65 | Extrahepatic | 3 | IIIB | 12.3 | / |
| BTC- 055-CE* | Italy | M | 74 | Intrahepatic | 2 | IIIB | 2.9 | / |
| 15T# | Spain | M | 68 | Extrahepatic | 1 | IIB | 2 | / |
| 16T# | Spain | M | 49 | Extrahepatic | 2 | IIB | 483 | / |
| 17T# | Spain | M | 71 | Extrahepatic | 2 | IIIA | 1692 | / |
| 18T# | Spain | M | 61 | Intrahepatic | 3 | IIIB | 827 | / |

Notes: *including also normal-matched samples; NA: not available; #: matched with bile samples analysed

**Table S3. Clinical characteristics of BTC and patients with benign biliary disease providing bile.**

| **Sample ID** | **Gender** | **Phenotype** | **Age at tumour diagnosis** | **Tumour location** | **Grade** | **Stage at diagnosis** | **CA 19-9 (UI/ml)** | **ERCP finding** | **Other clinical and pathological factors** |
| --- | --- | --- | --- | --- | --- | --- | --- | --- | --- |
| 1 | M | Control stenosis | 80 | / | / | / | 231 | Distal stricture | Chronic pancreatitis secondary to alcohol and tobacco. Transpapillary bile duct biopsy: non-specific acute and chronic inflammatory changes. |
| 2 | M | Control stenosis | 57 | / | / | / | 12000 | Perihilar stricture with hypoecogenic thickened bile duct wall. Choledocolithiasis | Repetead cholangitis and portal thrombosis. Bile duct brushing cytology: Atypical cells. Surgical specimen: bile duct inflammation. No tumour |
| 3 | M | Control stenosis | 66 | / | / | / | 684 | Middle and proximal common bile duct stricture | Portal vein thrombosis (right branch). Possible residual cholangitis. Bile duct brushing cytology: Negative to malignancy |
| 4 | M | Control stenosis | 59 | / | / | / | 35 | Distal stricture | Chronic pancreatitis secondary to alcohol and tobacco. Bile duct brushing cytology: Negative to malignancy |
| 11 | M | Control stenosis | 87 | / | / | / | 12000 | Distal stricture | Treated cholangitis. Bile duct brushing cytology: Reactive cells. Negative to malignancy |
| 5 | M | BTC | 84 | Extrahepatic | N/A | IIB | 66 | Irregular distal stricture | Extra and intrahepatic bile duct dilatation. Bile duct brushing cytology: Cholangiocarcinoma. |
| 6 | F | BTC | 67 | Extrahepatic | 2 | IIB | 100 | Irregular and large distal stricture | Surgical specimen: Middle and distal bile duct adenocarcinoma. R1 |
| 7 | F | BTC | 81 | Extrahepatic | 1 | IIB | 2 | Irregular distal stricture | Surgical specimen: Extrahepatic cholangiocarcinoma |
| 8 | F | BTC | 61 | Extrahepatic | 2 | IIB | 550 | Irregular distal stricture | Surgical specimen: Extrahepatic cholangiocarcinoma |
| 9 | M | BTC | 66 | Extrahepatic | 2 | II | 39 | Perihilar stricture | Surgical specimen: Cholangiocarcinoma |
| 10 | M | BTC | 81 | Extrahepatic | 2 | IV | 88 | Perihilar stricture | Fine-needle aspiration biopsy: Adenocarcinoma |
| 12 | F | BTC | 78 | Extrahepatic | N/A | I | 1347 | Distal stricture. Polypoid lesion | Common bile duct biopsy (cholangioscopy): intraductal papillary neoplasm. Predisposing history for BTC (PSC) |
| 13 | M | BTC | 84 | Extrahepatic | N/A | IIB | 12000 | Distal stricture | Bile duct brushing cytology: High-grade dysplasia |
| 14 | F | BTC | 70 | Intrahepatic | N/A | IV | 952819 | Intrahepatic stricture | Metastatic bile duct cancer with multiple liver lesions. Bile duct brushing cytology: non-diagnostic |
| 15 | M | BTC | 68 | Extrahepatic | 1 | IIB | 2 | Irregular distal stricture | Surgical specimen: cholangiocarcinoma |
| 16 | M | BTC | 49 | Extrahepatic | 2 | IIB | 483 | Irregular distal stricture | Surgical specimen: Intrapancreatic bile duct adenocarcinoma (1% sarcomatoid pattern) |
| 17 | M | BTC | 71 | Extrahepatic | 2 | IIIA | 1692 | Distal stricture | Surgical specimen: cholangiocarcinoma |
| 18 | M | BTC | 61 | Intrahepatic | 3 | IIIB | 827 | Multifocal irregular intrahepatic strictures | Surgical specimen: intrahepatic cholangiocarcinoma (sarcomatoid pattern) |
| 19 | M | BTC | 78 | Extrahepatic | 3 | IIA | 4 | Middle common bile duct stricture | Surgical specimen: cholangiocarcinoma |
| 20 | M | BTC | 73 | Extrahepatic | 2 | IIIA | 373 | Irregular perihilar stricture | Surgical specimen: extrahepatic cholangiocarcinoma |
| 21 | M | BTC | 62 | Extrahepatic | NA | IV | 6 | Distal stricture | Bile duct brushing cytology: adenocarcinoma. No surgery because of high-risk surgery patient. |
| 22 | M | BTC | 56 | Extrahepatic | NA | IV | 1478 | Perihilar stricture | Bile duct brushing cytology: adenocarcinoma. |
| 23 | M | BTC | 57 | Extrahepatic | NA | IV | 2 | Irregular perihilar stricture | Bile duct brushing cytology: adenocarcinoma. |
| 24 | F | BTC | 77 | Extrahepatic | NA | IV | 11 | Distal striciture | Fine-needle aspiration biopsy (hilar lymphadenopathy): Bile duct carcinoma metastasis |
| 25 | M | BTC | 60 | Intrahepatic | NA | IV | 243590 | Intrahepatic | Fine-needle aspiration liver mass biopsy: cholangiocarcinoma |
| 26 | F | BTC | 82 | Extrahepatic | NA | IV | 112 | Irregular Perihilar stricture | Bile duct brushing cytology: adenocarcinoma. |
| 27 | M | BTC | 57 | Extrahepatic | NA | IV | 2 | Irregular Perihilar stricture | Bile duct brushing cytology: adenocarcinoma. |
| 28 | F | BTC | 81 | Extrahepatic | NA | NA | 290 | Distal stricture | Bile duct brushing cytology: non-diagnostic. No surgery because of high-risk surgery patient. |
| 29 | M | BTC | 75 | Extrahepatic | 2 | I | 250 | Irregular distal stricture | Surgical specimen: cholangiocarcinoma |

Notes: NA: not available

Table S4. Methylation values of the 30 CGIs specifically altered in BTC in the Discovery and TCGA-CHOL datasets.

|  | **Discovery dataset** | | | | | **TCGA-CHOL dataset** | | | | |  | | | |
| --- | --- | --- | --- | --- | --- | --- | --- | --- | --- | --- | --- | --- | --- | --- |
| **CGI** | **β-value**  **Tumour** | **β-value**  **Normal** | **Δβ** | **combined.pval** | **combined.pval_adj** | **β-value**  **Tumour** | **β-value**  **Normal** | **Δβ** | **combined.pval** | **combined.pval_adj** | **Distance from TSS** | **Nearest**  **TSS gene** | **Distance from gene** | **Nearest**  **gene** |
| chr1:224805408-224805853 | 0.31 | 0.08 | 0.23 | 0.00127996 | 0.199131265 | 0.38 | 0.12 | 0.26 | 1.71E+09 | 0.00055159 | 1228 | *CNIH3* | 0 | *CNIH3* |
| chr10:111216604-111217083 | 0.33 | 0.11 | 0.22 | 0.00302071 | 0.236265951 | 0.43 | 0.12 | 0.31 | 0.0001045 | 0.002145251 | 426946 | *XPNPEP1* | 407440 | *XPNPEP1* |
| chr12:119212110-119212393 | 0.36 | 0.09 | 0.26 | 0.00298796 | 0.236265951 | 0.36 | 0.07 | 0.29 | 0.00051263 | 0.00717083 | 206906 | *SRRM4* | 206906 | *SRRM4* |
| chr12:75601081-75601752 | 0.33 | 0.13 | 0.21 | 0.00080747 | 0.195620171 | 0.50 | 0.09 | 0.41 | 1.69E+04 | 6.74E+06 | 1775 | *KCNC2* | 0 | *KCNC2* |
| chr17:46670522-46671458 | 0.33 | 0.09 | 0.23 | 0.00151295 | 0.214992741 | 0.30 | 0.08 | 0.22 | 0.00654375 | 0.049310432 | 0 | *HOXB5* | 0 | *HOXB-AS3* |
| chr17:46796234-46797292 | 0.36 | 0.11 | 0.25 | 0.00118229 | 0.197247818 | 0.51 | 0.13 | 0.38 | 3.98E+07 | 3.29E+08 | 2589 | *PRAC1* | 1789 | *PRAC1* |
| chr19:57182887-57183375 | 0.40 | 0.11 | 0.29 | 0.00314887 | 0.23907807 | 0.49 | 0.12 | 0.37 | 4.73E+08 | 0.000207481 | 0 | *ZNF835* | 0 | *ZNF835* |
| chr2:114260095-114261794 | 0.32 | 0.10 | 0.21 | 0.0038351 | 0.253266568 | 0.34 | 0.10 | 0.24 | 0.00383916 | 0.03260265 | 3433 | *FOXD4L1* | 1367 | *FOXD4L1* |
| chr2:176993479-176995557 | 0.36 | 0.09 | 0.27 | 0.00032765 | 0.192717559 | 0.48 | 0.07 | 0.41 | 3.16E+07 | 2.78E+09 | 0 | *HOXD8* | 0 | *HOXD8* |
| chr2:177027617-177028014 | 0.27 | 0.06 | 0.21 | 0.0039372 | 0.25476989 | 0.47 | 0.15 | 0.32 | 8.60E+09 | 0.001854012 | 790 | *HOXD3* | 790 | *HOXD3* |
| chr2:87088816-87089037 | 0.30 | 0.04 | 0.26 | 0.01919055 | 0.42657264 | 0.37 | 0.11 | 0.26 | 0.00015539 | 0.00292321 | 0 | *ANAPC1P1* | 0 | *RMND5A* |
| chr20:61703526-61704022 | 0.29 | 0.07 | 0.22 | 0.00061557 | 0.195620171 | 0.39 | 0.13 | 0.25 | 2.77E+09 | 0.000796178 | 28621 | *HAR1A* | 0 | *LINC01749* |
| chr21:27945010-27945646 | 0.29 | 0.08 | 0.21 | 0.00175614 | 0.215260795 | 0.46 | 0.10 | 0.35 | 1.45E+07 | 1.58E+09 | 0 | *CYYR1* | 0 | *CYYR1* |
| chr3:181437184-181437478 | 0.38 | 0.14 | 0.24 | 0.00452191 | 0.263195091 | 0.40 | 0.08 | 0.32 | 3.08E+09 | 0.000864514 | 7471 | *SOX2* | 0 | *SOX2-OT* |
| chr4:147576109-147576762 | 0.29 | 0.07 | 0.22 | 0.00054074 | 0.195620171 | 0.38 | 0.07 | 0.31 | 2.44E+09 | 0.000730616 | 16063 | *POU4F2* | 12485 | *POU4F2* |
| chr5:113696516-113699195 | 0.30 | 0.07 | 0.22 | 0.0019546 | 0.216448396 | 0.35 | 0.06 | 0.29 | 0.00012962 | 0.002532426 | 0 | *KCNN2* | 0 | *KCNN2* |
| chr5:140767196-140767695 | 0.33 | 0.12 | 0.21 | 0.01057561 | 0.341639376 | 0.38 | 0.12 | 0.27 | 0.0023245 | 0.022073389 | 0 | *PCDHGB4* | 0 | *PCDHGA1* |
| chr5:140777442-140777938 | 0.39 | 0.14 | 0.25 | 0.00494213 | 0.263255683 | 0.43 | 0.12 | 0.31 | 0.00037416 | 0.00566646 | 0 | *PCDHGB5* | 0 | *PCDHGA1* |
| chr5:145713641-145713913 | 0.33 | 0.11 | 0.22 | 0.0020246 | 0.218762425 | 0.44 | 0.13 | 0.31 | 2.95E+09 | 0.000835963 | 4673 | *POU4F3* | 4673 | *POU4F3* |
| chr5:76932317-76933523 | 0.33 | 0.10 | 0.23 | 0.00159148 | 0.214992741 | 0.35 | 0.13 | 0.22 | 0.00017372 | 0.003186954 | 998 | *OTP* | 0 | *OTP* |
| chr6:106433984-106434459 | 0.32 | 0.09 | 0.23 | 0.00588658 | 0.272404632 | 0.48 | 0.16 | 0.32 | 7.08E+07 | 5.05E+09 | 99735 | *PRDM1* | 99735 | *PRDM1* |
| chr6:150335525-150336278 | 0.29 | 0.07 | 0.22 | 0.00180826 | 0.215260795 | 0.40 | 0.11 | 0.29 | 0.000235 | 0.003978198 | 9244 | *RAET1K* | 4987 | *RAET1L* |
| chr6:27598687-27599146 | 0.35 | 0.12 | 0.23 | 0.00970143 | 0.328901873 | 0.33 | 0.06 | 0.27 | 0.00018872 | 0.003390078 | 62667 | *LINC01012* | 62667 | *LINC01012* |
| chr7:154001964-154002281 | 0.41 | 0.09 | 0.32 | 0.00067565 | 0.195620171 | 0.47 | 0.10 | 0.37 | 9.54E+08 | 0.000351354 | 65 | *DPP6* | 0 | *DPP6* |
| chr7:27135342-27136736 | 0.28 | 0.07 | 0.21 | 0.00106997 | 0.195620171 | 0.37 | 0.05 | 0.32 | 0.00021743 | 0.003759568 | 0 | *HOXA1* | 0 | *HOXA1* |
| chr7:27203915-27206462 | 0.37 | 0.12 | 0.25 | 0.00067286 | 0.195620171 | 0.56 | 0.18 | 0.38 | 6.68E+06 | 8.82E+08 | 0 | *HOXA9* | 0 | *HOXA10-HOXA9* |
| chr7:27208871-27209616 | 0.31 | 0.11 | 0.20 | 0.0228126 | 0.453729284 | 0.50 | 0.13 | 0.37 | 3.07E+08 | 0.000151232 | 0 | *MIR196B* | 0 | *HOXA10-HOXA9* |
| chr7:27283408-27283614 | 0.34 | 0.09 | 0.25 | 0.00272267 | 0.235646056 | 0.48 | 0.09 | 0.39 | 8.54E+07 | 0.00032581 | 1088 | *EVX1* | 0 | *EVX1* |
| chr8:24770908-24772547 | 0.37 | 0.09 | 0.28 | 0.00023564 | 0.19195134 | 0.40 | 0.15 | 0.25 | 0.00294713 | 0.02652519 | 0 | *NEFM* | 0 | *NEFM* |
| chr9:36739534-36739782 | 0.42 | 0.07 | 0.35 | 4.55E+09 | 0.187146817 | 0.35 | 0.12 | 0.23 | 0.00092069 | 0.010969813 | 83813 | *MIR4475* | 61853 | *MELK* |

**Table S5. CGIs showing an AUC ≥ 0.90 in the Discovery dataset and in TCGA-CHOL dataset.**

| **CGI** | **AUC**  **Discovery dataset** | **AUC**  **TCGA-CHOL dataset** |
| --- | --- | --- |
| chr10:111216604-111217083 | 0.92 | 0.93 |
| chr12:75601081-75601752 | 0.90 | 1.00 |
| chr17:46796234-46797292 | 0.93 | 0.94 |
| chr2:114260095-114261794 | 0.93 | 0.97 |
| chr2:176993479-176995557 | 0.92 | 0.95 |
| chr5:113696516-113699195 | 0.92 | 0.93 |
| chr7:27203915-27206462 | 0.90 | 1.00 |

Table S6. Methylation values of the 30 CGIs specifically altered in BTC in the GSE89803 dataset.

| **CGI** | **β-value**  **Tumour** | **β-value**  **Normal** | **Δβ** |
| --- | --- | --- | --- |
| chr1:224805408-224805853 | 0.39 | 0.13 | 0.26 |
| chr10:111216604-111217083 | 0.47 | 0.20 | 0.27 |
| chr12:119212110-119212393 | 0.42 | 0.14 | 0.28 |
| chr12:75601081-75601752 | 0.52 | 0.15 | 0.37 |
| chr17:46670522-46671458 | 0.30 | 0.07 | 0.23 |
| chr17:46796234-46797292 | 0.48 | 0.12 | 0.36 |
| chr19:57182887-57183375 | 0.52 | 0.13 | 0.39 |
| chr2:114260095-114261794 | 0.49 | 0.12 | 0.36 |
| chr2:176993479-176995557 | 0.53 | 0.12 | 0.41 |
| chr2:177027617-177028014* | 0.45 | 0.30 | 0.15 |
| chr2:87088816-87089037 | 0.34 | 0.13 | 0.21 |
| chr20:61703526-61704022 | 0.42 | 0.17 | 0.25 |
| chr21:27945010-27945646 | 0.50 | 0.13 | 0.37 |
| chr3:181437184-181437478 | 0.37 | 0.04 | 0.34 |
| chr4:147576109-147576762 | 0.43 | 0.15 | 0.28 |
| chr5:113696516-113699195 | 0.40 | 0.08 | 0.32 |
| chr5:140767196-140767695 | 0.40 | 0.12 | 0.29 |
| chr5:140777442-140777938 | 0.43 | 0.15 | 0.28 |
| chr5:145713641-145713913 | 0.44 | 0.13 | 0.31 |
| chr5:76932317-76933523 | 0.37 | 0.11 | 0.26 |
| chr6:106433984-106434459* | 0.52 | 0.44 | 0.08 |
| chr6:150335525-150336278 | 0.39 | 0.13 | 0.27 |
| chr6:27598687-27599146* | 0.25 | 0.06 | 0.19 |
| chr7:154001964-154002281 | 0.47 | 0.13 | 0.34 |
| chr7:27135342-27136736 | 0.42 | 0.04 | 0.39 |
| chr7:27203915-27206462 | 0.54 | 0.15 | 0.39 |
| chr7:27208871-27209616 | 0.45 | 0.14 | 0.31 |
| chr7:27283408-27283614 | 0.37 | 0.07 | 0.29 |
| chr8:24770908-24772547 | 0.46 | 0.14 | 0.32 |
| chr9:36739534-36739782 | 0.38 | 0.09 | 0.30 |

Notes: *CGIs not differentially methylated

**Table S7. Clinical characteristics of TCGA-CHOL patients.**

| **bcr_patient_barcode** | **ID** | **Sample type** | **Tumour location** | **Grade** | **Stage** |
| --- | --- | --- | --- | --- | --- |
| TCGA-3X-AAV9 | TCGA-3X-AAV9-01A-72D-A418-05 | Tumour | Intrahepatic | 2 | I |
| TCGA-3X-AAVA | TCGA-3X-AAVA-01A-11D-A418-05 | Tumour | Intrahepatic | 2 | II |
| TCGA-3X-AAVB | TCGA-3X-AAVB-01A-31D-A418-05 | Tumour | Extrahepatic | 1 | IV |
| TCGA-3X-AAVC | TCGA-3X-AAVC-01A-21D-A418-05 | Tumour | Hilar/perihilar | 3 | I |
| TCGA-3X-AAVE | TCGA-3X-AAVE-01A-11D-A418-05 | Tumour | Intrahepatic | 2 | II |
| TCGA-4G-AAZO | TCGA-4G-AAZO-01A-12D-A418-05 | Tumour | Intrahepatic | 2 | II |
| TCGA-4G-AAZT | TCGA-4G-AAZT-01A-11D-A418-05 | Tumour | Intrahepatic | 2 | I |
| TCGA-W5-AA2G | TCGA-W5-AA2G-01A-11D-A418-05 | Tumour | Intrahepatic | 3 | I |
| TCGA-W5-AA2H | TCGA-W5-AA2H-01A-31D-A418-05 | Tumour | Extrahepatic | 3 | III |
| TCGA-W5-AA2I | TCGA-W5-AA2I-01A-32D-A418-05 | Tumour | Intrahepatic | 2 | I |
| TCGA-W5-AA2I | TCGA-W5-AA2I-11A-11D-A418-05 | Normal |  |  |  |
| TCGA-W5-AA2O | TCGA-W5-AA2O-01A-11D-A418-05 | Tumour | Intrahepatic | 3 | I |
| TCGA-W5-AA2Q | TCGA-W5-AA2Q-01A-11D-A418-05 | Tumour | Intrahepatic | 2 | II |
| TCGA-W5-AA2Q | TCGA-W5-AA2Q-11A-11D-A418-05 | Normal |  |  |  |
| TCGA-W5-AA2R | TCGA-W5-AA2R-01A-11D-A418-05 | Tumour | Intrahepatic | 3 | I |
| TCGA-W5-AA2R | TCGA-W5-AA2R-11A-11D-A418-05 | Normal |  |  |  |
| TCGA-W5-AA2T | TCGA-W5-AA2T-01A-12D-A418-05 | Tumour | Intrahepatic | 3 | II |
| TCGA-W5-AA2U | TCGA-W5-AA2U-01A-11D-A418-05 | Tumour | Intrahepatic | 3 | I |
| TCGA-W5-AA2U | TCGA-W5-AA2U-11A-11D-A418-05 | Normal |  |  |  |
| TCGA-W5-AA2W | TCGA-W5-AA2W-01A-11D-A418-05 | Tumour | Intrahepatic | 3 | IV |
| TCGA-W5-AA2X | TCGA-W5-AA2X-01A-11D-A418-05 | Tumour | Hilar/perihilar | 4 | IV |
| TCGA-W5-AA2X | TCGA-W5-AA2X-11A-11D-A418-05 | Normal |  |  |  |
| TCGA-W5-AA2Z | TCGA-W5-AA2Z-01A-11D-A418-05 | Tumour | Intrahepatic | 3 | II |
| TCGA-W5-AA30 | TCGA-W5-AA30-01A-31D-A418-05 | Tumour | Intrahepatic | 3 | I |
| TCGA-W5-AA30 | TCGA-W5-AA30-11A-11D-A418-05 | Normal |  |  |  |
| TCGA-W5-AA31 | TCGA-W5-AA31-01A-11D-A418-05 | Tumour | Intrahepatic | 3 | I |
| TCGA-W5-AA31 | TCGA-W5-AA31-11A-11D-A418-05 | Normal |  |  |  |
| TCGA-W5-AA33 | TCGA-W5-AA33-01A-11D-A418-05 | Tumour | Intrahepatic | 3 | I |
| TCGA-W5-AA34 | TCGA-W5-AA34-01A-11D-A418-05 | Tumour | Intrahepatic | 2 | I |
| TCGA-W5-AA34 | TCGA-W5-AA34-11A-11D-A418-05 | Normal |  |  |  |
| TCGA-W5-AA36 | TCGA-W5-AA36-01A-11D-A418-05 | Tumour | Hilar/perihilar | 3 | IV |
| TCGA-W5-AA38 | TCGA-W5-AA38-01A-11D-A418-05 | Tumour | Intrahepatic | 3 | I |
| TCGA-W5-AA39 | TCGA-W5-AA39-01A-11D-A418-05 | Tumour | Intrahepatic | 4 | II |
| TCGA-W6-AA0S | TCGA-W6-AA0S-01A-11D-A418-05 | Tumour | Intrahepatic | 2 | I |
| TCGA-WD-A7RX | TCGA-WD-A7RX-01A-12D-A418-05 | Tumour | Intrahepatic | 2 | II |
| TCGA-YR-A95A | TCGA-YR-A95A-01A-12D-A418-05 | Tumour | Hilar/perihilar | 2 | IV |
| TCGA-ZD-A8I3 | TCGA-ZD-A8I3-01A-11D-A418-05 | Tumour | Intrahepatic | 3 | II |
| TCGA-ZH-A8Y1 | TCGA-ZH-A8Y1-01A-11D-A418-05 | Tumour | Intrahepatic | 2 | IV |
| TCGA-ZH-A8Y2 | TCGA-ZH-A8Y2-01A-11D-A418-05 | Tumour | Intrahepatic | 2 | I |
| TCGA-ZH-A8Y4 | TCGA-ZH-A8Y4-01A-11D-A418-05 | Tumour | Intrahepatic | 3 | I |
| TCGA-ZH-A8Y5 | TCGA-ZH-A8Y5-01A-11D-A418-05 | Tumour | Intrahepatic | 3 | IV |
| TCGA-ZH-A8Y6 | TCGA-ZH-A8Y6-01A-11D-A418-05 | Tumour | Intrahepatic | 2 | I |
| TCGA-ZH-A8Y8 | TCGA-ZH-A8Y8-01A-51D-A418-05 | Tumour | Intrahepatic | 2 | I |
| TCGA-ZU-A8S4 | TCGA-ZU-A8S4-01A-11D-A418-05 | Tumour | Intrahepatic | 3 | I |
| TCGA-ZU-A8S5 | TCGA-ZU-A8S4-11A-11D-A418-05 | Normal |  |  |  |

**Table S8. Clinical characteristics of GSE89803 patients.**

| **Sample ID** | **Collection center** | **Sample type** | **Gender** | **Age at surgery** | **Type (Fluke-Pos/Fluke-Neg)** | **Localization** | **Stage** |
| --- | --- | --- | --- | --- | --- | --- | --- |
| CCA_SG_28 | Singapore | Tumour | F | 67 | Fluke-Neg | Intrahepatic | I |
| CCA_SG_10 | Singapore | Tumour | M | 74 | Fluke-Neg | Intrahepatic | I |
| CCA_RO_15 | Romania | Tumour | F | 61 | Fluke-Neg | Perihilar | III |
| CCA_RO_5 | Romania | Tumour | F | 69 | Fluke-Neg | Intrahepatic | N/A |
| CCA_SG_25 | Singapore | Tumour | F | 74 | Fluke-Neg | Intrahepatic | I |
| CCA_RO_2 | Romania | Tumour | F | 49 | Fluke-Neg | Perihilar | IV |
| CCA_SG_12 | Singapore | Tumour | F | 61 | Fluke-Neg | Intrahepatic | I |
| CCA_RO_46 | Romania | Tumour | M | 69 | Fluke-Neg | Extrahepatic | II |
| CCA_SG_6 | Singapore | Tumour | M | 51 | Fluke-Neg | Intrahepatic | I |
| CCA_RO_8 | Romania | Tumour | M | 47 | Fluke-Neg | Perihilar | II |
| CCA_SG_49 | Singapore | Tumour | M | 49 | Fluke-Neg | Perihilar | II |
| CCA_SG_9 | Singapore | Tumour | F | 60 | Fluke-Neg | Intrahepatic | IV |
| CCA_SG_14 | Singapore | Tumour | F | 61 | Fluke-Neg | Intrahepatic | N/A |
| CCA_SG_3 | Singapore | Tumour | F | 51 | Fluke-Neg | Intrahepatic | I |
| CCA_SG_15 | Singapore | Tumour | M | 53 | Fluke-Neg | Intrahepatic | III |
| CCA_SG_20 | Singapore | Tumour | M | 56 | Fluke-Neg | Intrahepatic | I |
| CCA_SG_16 | Singapore | Tumour | M | 55 | Fluke-Neg | Intrahepatic | III |
| CCA_SG_48 | Singapore | Tumour | F | 77 | Fluke-Neg | Perihilar | N/A |
| CCA_TH_18 | Thailand | Tumour | M | 56 | Fluke-Pos | Intrahepatic | IV |
| CCA_SG_1 | Singapore | Tumour | F | 73 | Fluke-Neg | Intrahepatic | III |
| CCA_TH_17 | Thailand | Tumour | M | 66 | Fluke-Pos | Intrahepatic | III |
| CCA_SG_11 | Singapore | Tumour | F | 69 | Fluke-Neg | Intrahepatic | IV |
| CCA_TH_14 | Thailand | Tumour | F | 64 | Fluke-Pos | Intrahepatic | III |
| CCA_SG_2 | Singapore | Tumour | F | 67 | Fluke-Neg | Intrahepatic | IV |
| CCA_TH_131 | Thailand | Tumour | M | 52 | Fluke-Pos | Perihilar | III |
| CCA_SG_4 | Singapore | Tumour | M | 61 | Fluke-Neg | Intrahepatic | I |
| CCA_TH_19 | Thailand | Tumour | F | 65 | Fluke-Pos | Intrahepatic | III |
| CCA_TH_120 | Thailand | Tumour | F | 45 | Fluke-Pos | Intrahepatic | IV |
| CCA_TH_122 | Thailand | Tumour | M | 50 | Fluke-Pos | Intrahepatic | IV |
| CCA_TH_55 | Thailand | Tumour | M | 73 | Fluke-Pos | Intrahepatic | III |
| CCA_TH_9 | Thailand | Tumour | M | 48 | Fluke-Pos | Intrahepatic | III |
| CCA_TH_28 | Thailand | Tumour | F | 52 | Fluke-Pos | Intrahepatic | IV |
| CCA_TH_114 | Thailand | Tumour | F | 49 | Fluke-Pos | Perihilar | III |
| CCA_TH_3 | Thailand | Tumour | F | 76 | Fluke-Pos | Perihilar | III |
| CCA_TH_15 | Thailand | Tumour | M | 37 | Fluke-Pos | Perihilar | III |
| CCA_TH_43 | Thailand | Tumour | F | 38 | Fluke-Pos | Intrahepatic | IV |
| CCA_TH_23 | Thailand | Tumour | F | 56 | Fluke-Pos | Perihilar | I |
| CCA_TH_5 | Thailand | Tumour | M | 60 | Fluke-Pos | Perihilar | IV |
| CCA_TH_1 | Thailand | Tumour | F | 49 | Fluke-Pos | Intrahepatic | III |
| CCA_TH_47 | Thailand | Tumour | M | 66 | Fluke-Pos | Perihilar | 0 |
| CCA_TH_27 | Thailand | Tumour | F | 72 | Fluke-Pos | Intrahepatic | IV |
| CCA_TH_50 | Thailand | Tumour | M | 60 | Fluke-Pos | Perihilar | III |
| CCA_TH_69 | Thailand | Tumour | F | 48 | Fluke-Pos | Intrahepatic | II |
| CCA_RO_42 | Romania | Tumour | M | 70 | Fluke-Neg | Extrahepatic | II |
| CCA_TH_72 | Thailand | Tumour | M | 66 | Fluke-Pos | Perihilar | II |
| CCA_RO_38 | Romania | Tumour | F | 44 | Fluke-Neg | Intrahepatic | IV |
| CCA_TH_81 | Thailand | Tumour | M | 49 | Fluke-Pos | Perihilar | IV |
| CCA_RO_3 | Romania | Tumour | M | 46 | Fluke-Neg | Perihilar | II |
| CCA_TH_85 | Thailand | Tumour | F | 52 | Fluke-Pos | Intrahepatic | II |
| CCA_RO_19 | Romania | Tumour | F | 66 | Fluke-Neg | Intrahepatic | N/A |
| CCA_RO_10 | Romania | Tumour | F | 61 | Fluke-Neg | Intrahepatic | III |
| CCA_RO_43 | Romania | Tumour | M | 40 | Fluke-Neg | Intrahepatic | IV |
| CCA_TH_121 | Thailand | Tumour | M | 70 | Fluke-Pos | Intrahepatic | IV |
| CCA_TH_65 | Thailand | Tumour | M | 42 | Fluke-Pos | Intrahepatic | IV |
| CCA_TH_132 | Thailand | Tumour | M | 57 | Fluke-Pos | Intrahepatic | IV |
| CCA_TH_10 | Thailand | Tumour | F | 69 | Fluke-Pos | Intrahepatic | IV |
| CCA_TH_127 | Thailand | Tumour | F | 51 | Fluke-Pos | Perihilar | IV |
| CCA_TH_67 | Thailand | Tumour | M | 52 | Fluke-Pos | Perihilar | IV |
| CCA_TH_20 | Thailand | Tumour | M | 63 | Fluke-Pos | Intrahepatic | IV |
| CCA_TH_75 | Thailand | Tumour | M | 52 | Fluke-Pos | Intrahepatic | III |
| CCA_TH_58 | Thailand | Tumour | M | 63 | Fluke-Pos | Perihilar | III |
| CCA_TH_78 | Thailand | Tumour | F | 60 | Fluke-Pos | Intrahepatic | IV |
| CCA_TH_59 | Thailand | Tumour | M | 47 | Fluke-Pos | Intrahepatic | III |
| CCA_SG_5 | Singapore | Tumour | F | 78 | Fluke-Neg | Intrahepatic | IV |
| CCA_RO_44 | Romania | Tumour | F | 61 | Fluke-Neg | Intrahepatic | IV |
| CCA_SG_24 | Singapore | Tumour | F | 45 | Fluke-Neg | Perihilar | IV |
| CCA_RO_35 | Romania | Tumour | M | 62 | Fluke-Neg | Intrahepatic | N/A |
| CCA_SG_51 | Singapore | Tumour | M | 63 | Fluke-Neg | Intrahepatic | II |
| CCA_SG_52 | Singapore | Tumour | F | 26 | Fluke-Neg | Intrahepatic | II |
| CCA_SG_34 | Singapore | Tumour | M | 35 | Fluke-Neg | Perihilar | III |
| CCA_SG_7 | Singapore | Tumour | M | 68 | Fluke-Neg | Extrahepatic | I |
| CCA_SG_50 | Singapore | Tumour | M | 32 | Fluke-Neg | Intrahepatic | IV |
| CCA_SG_53 | Singapore | Tumour | M | 47 | Fluke-Neg | Perihilar | II |
| CCA_SG_8 | Singapore | Tumour | M | 57 | Fluke-Neg | Intrahepatic | I |
| CCA_TH_123 | Thailand | Tumour | M | 62 | Fluke-Pos | Intrahepatic | IV |
| CCA_TH_21 | Thailand | Tumour | F | 40 | Fluke-Pos | Intrahepatic | I |
| CCA_TH_128 | Thailand | Tumour | M | 55 | Fluke-Pos | Perihilar | IV |
| CCA_TH_22 | Thailand | Tumour | F | 56 | Fluke-Pos | Intrahepatic | II |
| CCA_TH_2 | Thailand | Tumour | M | 46 | Fluke-Pos | Perihilar | III |
| CCA_TH_31 | Thailand | Tumour | M | 55 | Fluke-Pos | Intrahepatic | IV |
| CCA_TH_4 | Thailand | Tumour | M | 53 | Fluke-Pos | Perihilar | IV |
| CCA_TH_44 | Thailand | Tumour | M | 64 | Fluke-Pos | Perihilar | IV |
| CCA_TH_130 | Thailand | Tumour | M | 51 | Fluke-Pos | Perihilar | N/A |
| CCA_TH_6 | Thailand | Tumour | F | 51 | Fluke-Pos | Intrahepatic | IV |
| CCA_TH_7 | Thailand | Tumour | M | 61 | Fluke-Pos | Perihilar | III |
| CCA_TH_8 | Thailand | Tumour | F | 53 | Fluke-Pos | Intrahepatic | III |
| CCA_TH_11 | Thailand | Tumour | F | 79 | Fluke-Pos | Intrahepatic | IV |
| CCA_TH_12 | Thailand | Tumour | M | 52 | Fluke-Pos | Perihilar | III |
| CCA_TH_13 | Thailand | Tumour | M | 57 | Fluke-Pos | Perihilar | III |
| CCA_TH_79 | Thailand | Tumour | M | 64 | Fluke-Pos | Intrahepatic | IV |
| CCA_SG_21 | Singapore | Tumour | F | 55 | Fluke-Neg | Intrahepatic | IV |
| CCA_SG_54 | Singapore | Tumour | F | 60 | Fluke-Neg | Intrahepatic | IV |
| CCA_SG_13 | Singapore | Tumour | M | 60 | Fluke-Neg | Intrahepatic | I |
| CCA_SG_36 | Singapore | Tumour | F | 59 | Fluke-Neg | Intrahepatic | II |
| CCA_SG_17 | Singapore | Tumour | F | 77 | Fluke-Neg | Intrahepatic | IV |
| CCA_SG_18 | Singapore | Tumour | F | 71 | Fluke-Neg | Extrahepatic | II |
| CCA_SG_19 | Singapore | Tumour | M | 75 | Fluke-Neg | Intrahepatic | II |
| CCA_SG_29 | Singapore | Tumour | F | 39 | Fluke-Neg | Intrahepatic | I |
| CCA_RO_7 | Romania | Tumour | M | 68 | Fluke-Neg | Extrahepatic | I |
| CCA_RO_29 | Romania | Tumour | M | 72 | Fluke-Neg | Perihilar | II |
| CCA_RO_45 | Romania | Tumour | M | 69 | Fluke-Neg | Intrahepatic | NA |
| CCA_RO_25 | Romania | Tumour | M | 78 | Fluke-Neg | Intrahepatic | N/A |
| CCA_RO_4 | Romania | Tumour | F | 53 | Fluke-Neg | Intrahepatic | N/A |
| CCA_RO_6 | Romania | Tumour | M | N/A | Fluke-Neg | Intrahepatic | N/A |
| CCA_RO_23 | Romania | Tumour | F | 53 | Fluke-Neg | Perihilar | III |
| CCA_RO_31 | Romania | Tumour | M | 58 | Fluke-Neg | Extrahepatic | II |
| CCA_IT_1 | Italy | Tumour | F | 69 | Fluke-Neg | Intrahepatic | IV |
| CCA_IT_2 | Italy | Tumour | F | 76 | Fluke-Neg | Intrahepatic | III |
| CCA_IT_3 | Italy | Tumour | M | 53 | Fluke-Neg | Intrahepatic | IV |
| CCA_IT_4 | Italy | Tumour | M | 73 | Fluke-Neg | Intrahepatic | III |
| CCA_BR_1 | Brazil | Tumour | M | 53 | Fluke-Neg | Intrahepatic | N/A |
| CCA_BR_2 | Brazil | Tumour | F | 49 | Fluke-Neg | Intrahepatic | IV |
| CCA_KR_1 | Korea | Tumour | M | 63 | Fluke-Neg | Intrahepatic | N/A |
| CCA_KR_2 | Korea | Tumour | M | 70 | Fluke-Neg | Intrahepatic | N/A |
| CCA_FR_13 | France | Tumour | M | 51 | Fluke-Neg | Intrahepatic | N/A |
| CCA_FR_11 | France | Tumour | M | 45 | Fluke-Neg | Intrahepatic | II |
| CCA_FR_10 | France | Tumour | M | 29 | Fluke-Neg | Intrahepatic | IV |
| CCA_FR_9 | France | Tumour | M | 62 | Fluke-Neg | Intrahepatic | IV |
| CCA_FR_8 | France | Tumour | F | 73 | Fluke-Neg | Intrahepatic | N/A |
| CCA_FR_3 | France | Tumour | F | 60 | Fluke-Neg | Intrahepatic | N/A |
| CCA_FR_4 | France | Tumour | M | 75 | Fluke-Neg | Intrahepatic | IV |
| CCA_FR_5 | France | Tumour | F | 76 | Fluke-Neg | Intrahepatic | N/A |
| CCA_FR_6 | France | Tumour | M | 68 | Fluke-Neg | Intrahepatic | I |
| CCA_FR_7 | France | Tumour | F | 77 | Fluke-Neg | Intrahepatic | IV |
| CCA_FR_2 | France | Tumour | M | 78 | Fluke-Neg | Intrahepatic | N/A |
| CCA_FR_1 | France | Tumour | F | 49 | Fluke-Neg | Intrahepatic | N/A |
| CCA_FR_15 | France | Tumour | M | 56 | Fluke-Neg | Intrahepatic | IV |
| CCA_FR_16 | France | Tumour | F | 78 | Fluke-Neg | Intrahepatic | N/A |
| CCA_FR_14 | France | Tumour | F | 52 | Fluke-Neg | Intrahepatic | N/A |
| CCA_FR_12 | France | Tumour | F | 43 | Fluke-Neg | Intrahepatic | IV |
| CCA_CH_5 | China | Tumour | M | 75 | Fluke-Neg | Intrahepatic | I |
| CCA_CH_6 | China | Tumour | F | 59 | Fluke-Neg | Intrahepatic | III |
| CCA_CH_1 | China | Tumour | F | 49 | Fluke-Neg | Perihilar | II |
| CCA_CH_7 | China | Tumour | F | 71 | Fluke-Neg | Intrahepatic | IV |
| CCA_CH_2 | China | Tumour | F | 61 | Fluke-Pos | Perihilar | II |
| CCA_CH_8 | China | Tumour | M | 60 | Fluke-Neg | Intrahepatic | II |
| CCA_CH_3 | China | Tumour | M | 46 | Fluke-Neg | Perihilar | II |
| CCA_CH_4 | China | Tumour | M | 46 | Fluke-Neg | Extrahepatic | III |
| NBD_SG_3 |  | Normal |  |  |  |  |  |
| NBD_SG_1 |  | Normal |  |  |  |  |  |
| NBD_SG_2 |  | Normal |  |  |  |  |  |
| NBD_SG_4 |  | Normal |  |  |  |  |  |

**Supplementary Figures**


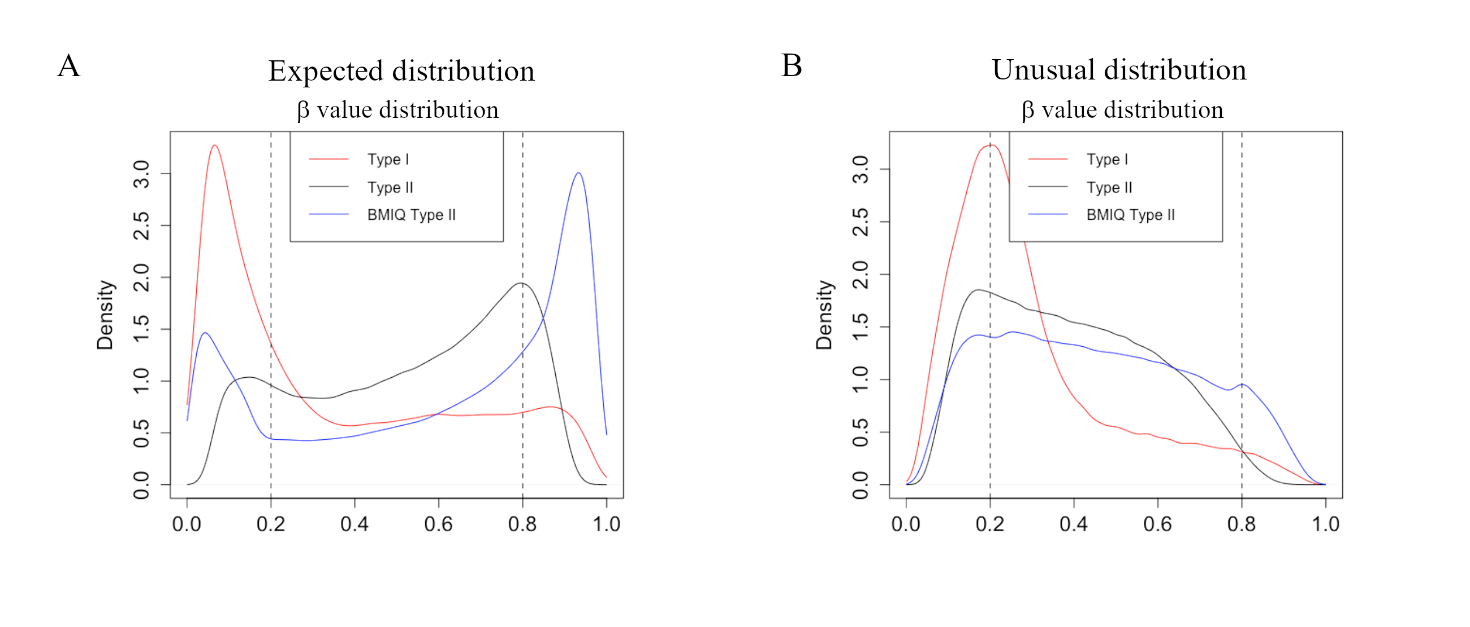


**Fig. S**1. Visualization of β-value density distribution of exemplificative samples. (A and B) Example of an expected (A) and unusual (B) β-value density distribution


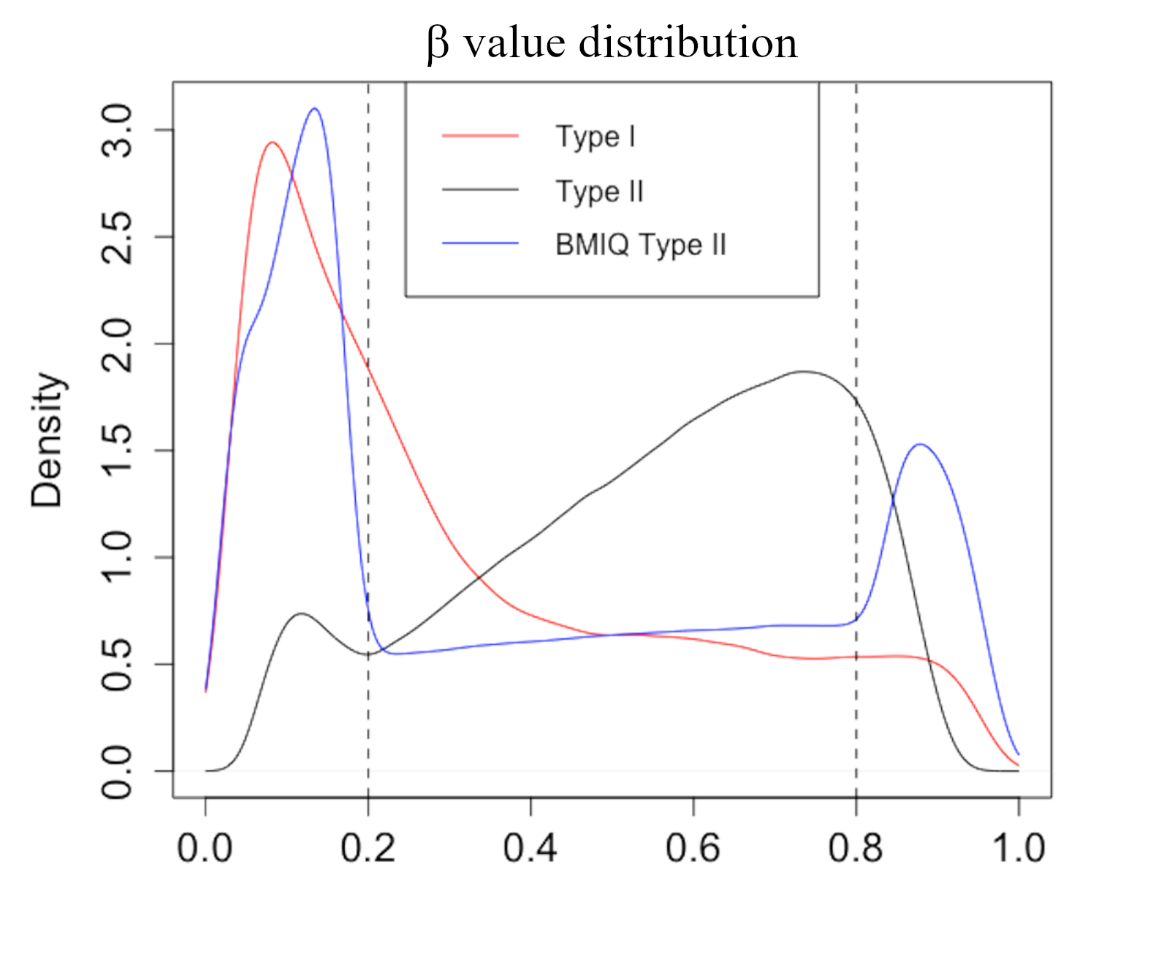


Fig. S2. β-value density distribution for the sample censored in ddPCR results.

**
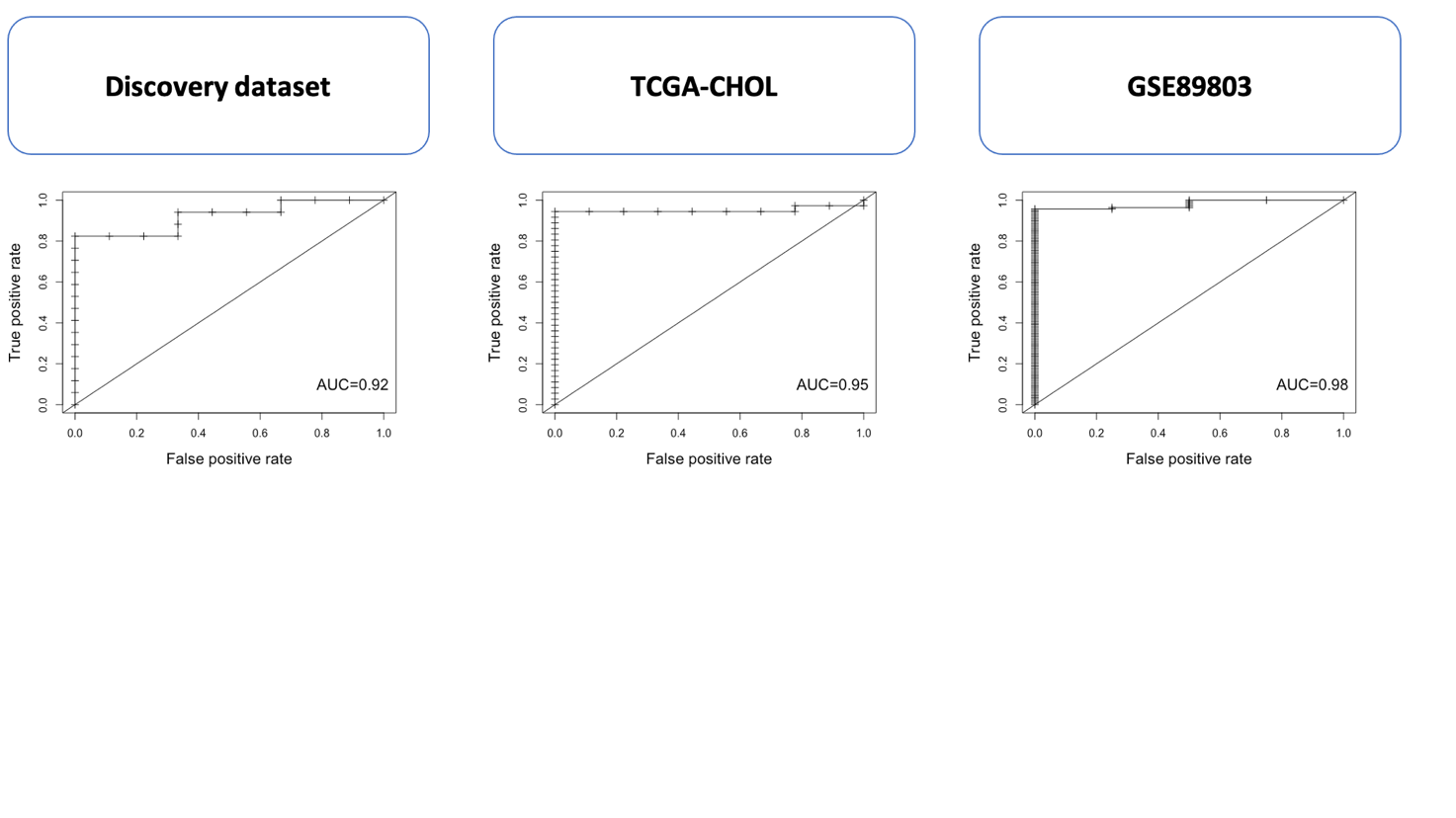
**

Fig. S3. ROC curves relative to CGI chr2:176993479-176995557 in the Discovery, TCGA-CHOL and GSE89803 datasets.


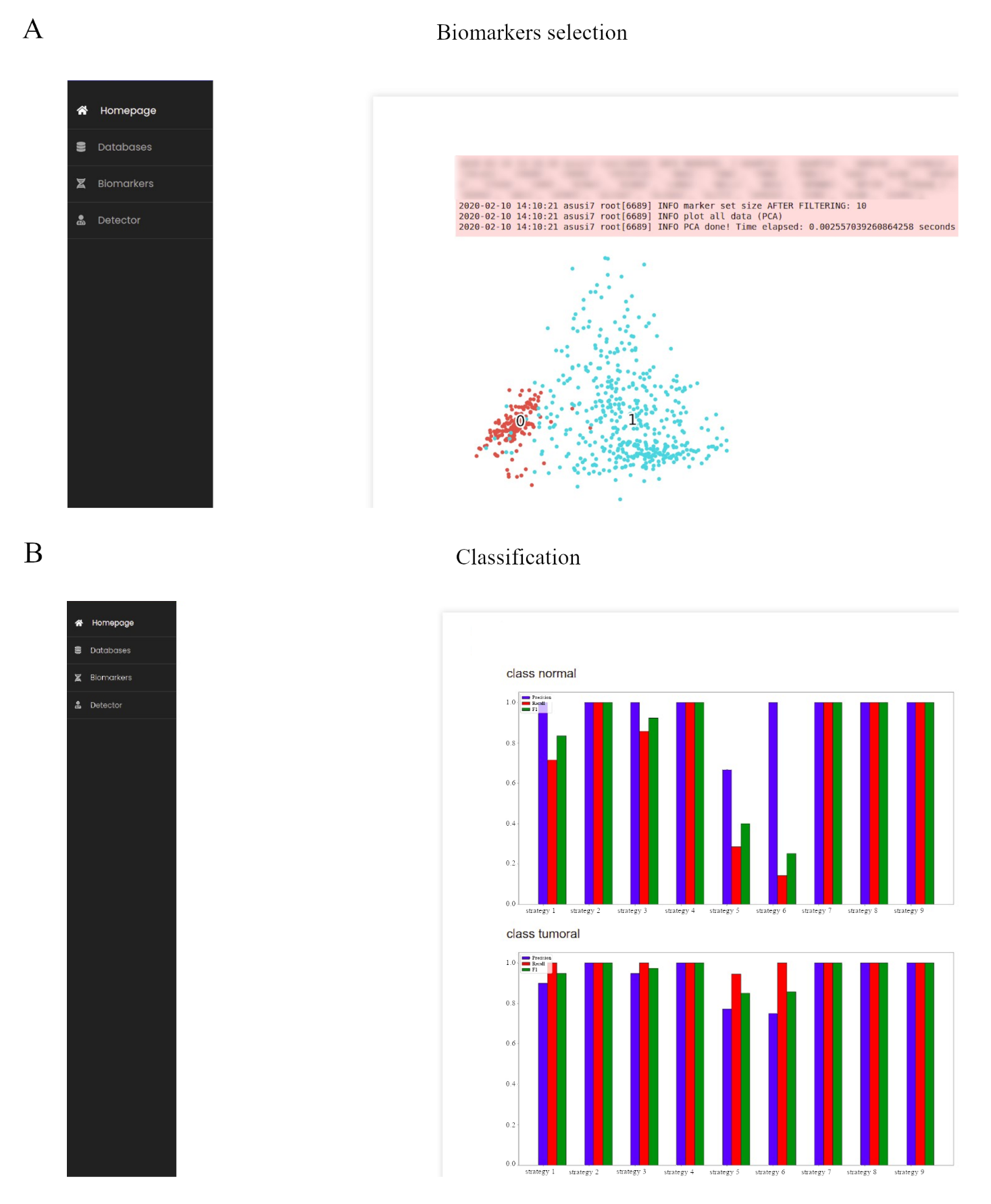
 **F**ig. S4. Example of biomarker selection and classification procedures applied by the algorithm. (A) Biomarkers selection. (B) Classification

**Supplementary Document S1**

**Supplementary Methods**

**Statistical power calculation**

Genome-wide methylation study

Statistical power was calculated similarly to previous epigenome-wide study (EWAS) reports ^1^. In contrast with our study, most EWAS analyse methylation in blood samples, which facilitates the recruitment of donors. The main limiting factor of our study was the number of normal (control) samples, due to the difficulty of obtaining biliary non-tumour tissues from BTC patients or healthy donors. Statistical power is greatly influenced by the intra-group variability of the measurement. To estimate the expected CpG methylation variability in our samples, we analysed the β-values standard deviation (SD) of normal and tumour samples from the TCGA-CHOL dataset (Supplementary Fig. S5). The β-value distribution is restricted to the interval [0,1], with a theoretical upper limit of SD ≤ 0.5. In practice, autosome CpG site methylation from homogeneous samples typically exhibits a much smaller variability. CpG methylation exhibited very low variability in normal samples (median SD=0.02, 90th percentile = 0.09). In tumour samples, the variability was significantly higher reflecting higher inter-sample heterogeneity (median SD=0.09, 90th percentile = 0.24).

**
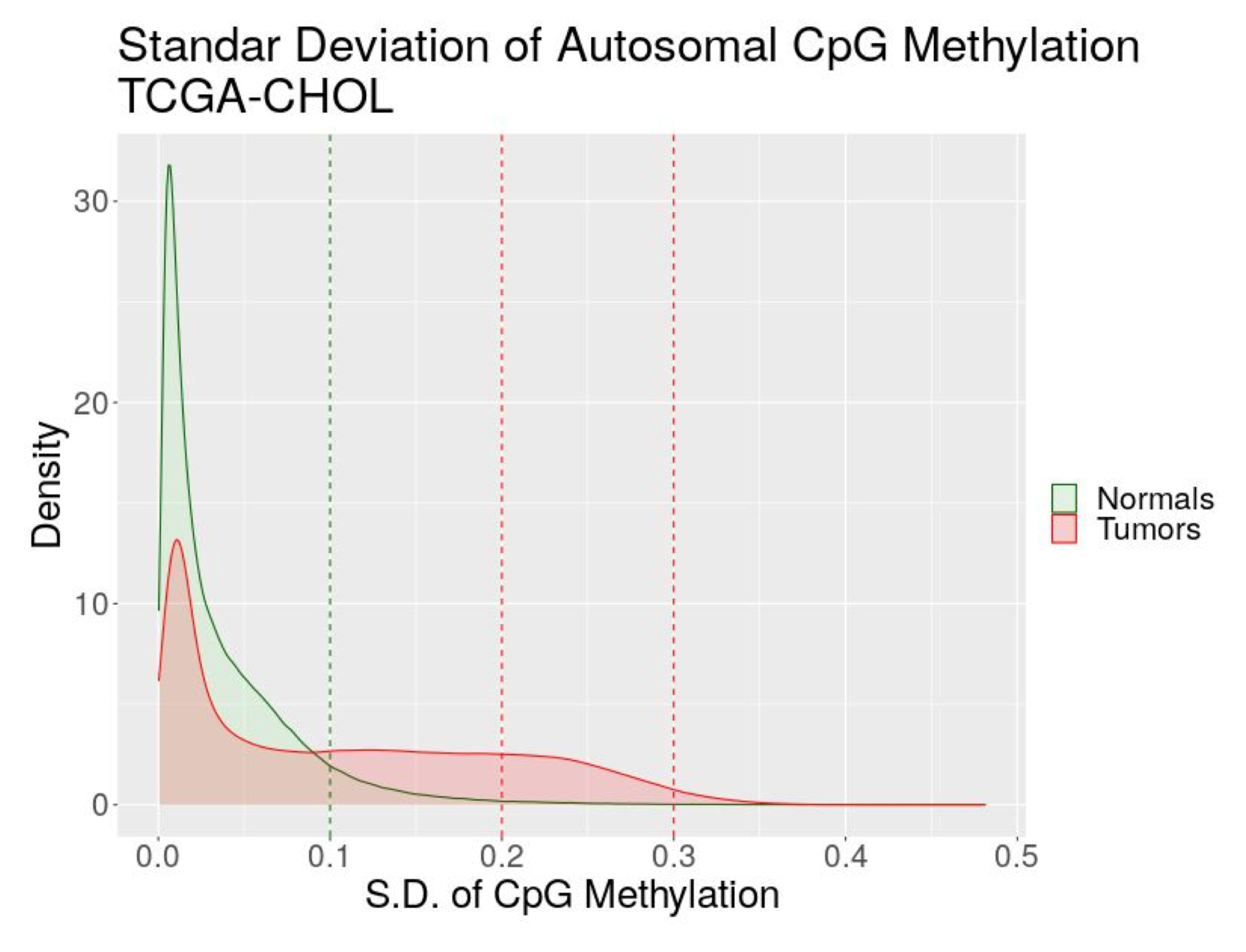
Fig. S5**. **Distribution of the standard deviation of CpG methylation probes from the TCGA-CHOL dataset.** Only autosomal probes were considered. In green, normal samples (*N* =9). In red, tumour samples (*N* =3 6). Vertical dashed lines indicate the standard deviation values employed in the statistical power calculations of our study.

Statistical power calculations for the difference in mean methylation between normal and tumours were performed applying the t-student power methods implemented in the pwr R package (<https://cran.r-project.org/package=pwr>), considering an unbalanced design (10 controls and a variable number of tumour samples), a single locus significance threshold of *P*-value=0.01 and a genome-wide significance threshold of *P*-value =1×10^−6^ ^1^. We considered a SD=0.1 for both normal and tumour samples.


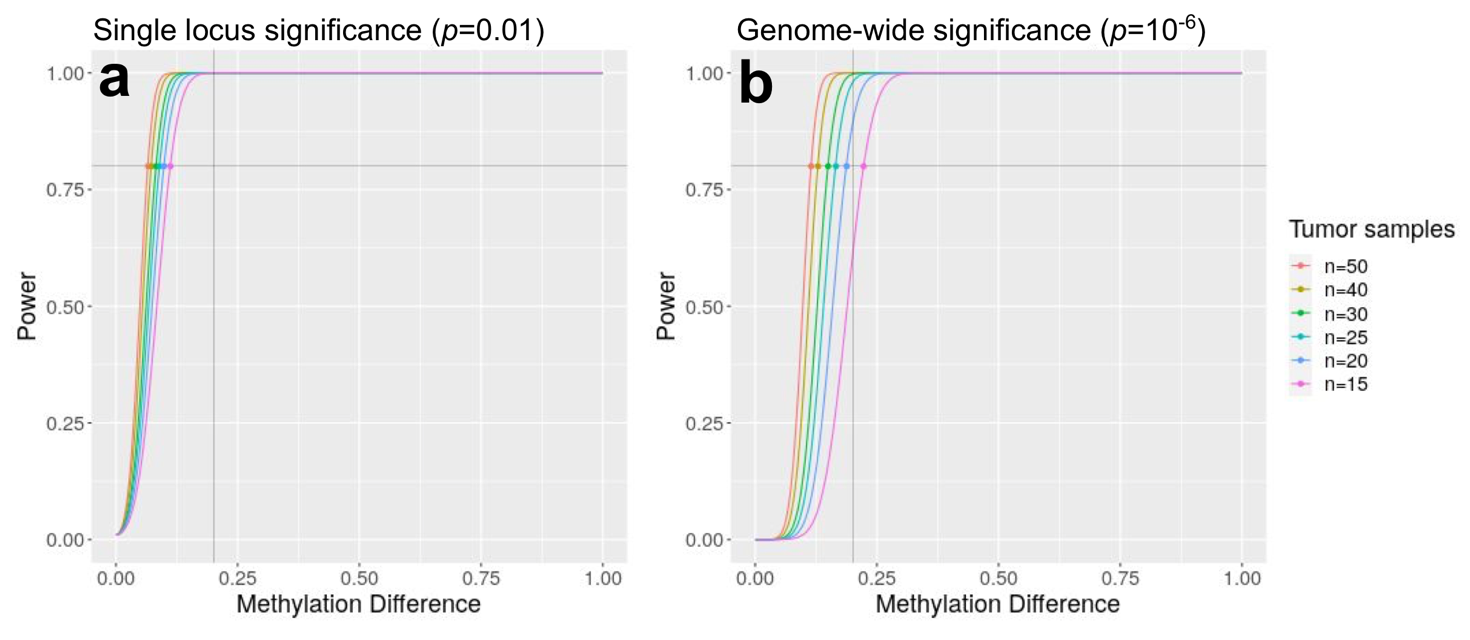


**Fig. S6**. **Statistical power calculations for the difference in mean methylation between normal and tumours for a single locus (a) and genome-wide (b) significance.** Number of normal samples was fixed at 10, and the number of tumour samples was variable between 15 and 50, considering a SD of 0.1. The horizontal grey line indicates a statistical power of 0.8. The vertical grey line indicates a methylation difference of |Δβ| = 0.2, frequently considered as the lower limit of a biologically relevant difference in methylation.

Under these assumptions, a study with a limited number of normal samples (*N* =10) would be able to identify biologically relevant differences in methylation (|Δβ| = 0.2) at genome-wide significance with as little as 20 tumour samples if those alterations occur in most tumour samples (thus with a low SD) (Supplementary Fig. S6).

Validation study by ddPCR

Sample size and statistical power calculations for Fisher’s exact tests were performed using the exact2x2 package in R ^2^.

Considering the sensitivity of the two-biomarker panel (chr2:176993479-176995557, chr5:145713641-145713913) on the TCGA validation cohort (0.944, slightly lower than 0.964 found in the GSE89803 validation dataset), a specificity of 1 found in both the TCGA and GSE89803 cohorts, and a type-I error probability of α=0.01, the minimum sample size to achieve statistical power over 80% in Fisher’s exact tests was *N* =7 for both the normal and the tumour tissues. Considering the sample size of our validation tissue dataset (18 BTC and 14 normal tissues) and bile samples dataset (24 from BTC patients and 5 from patients with benign biliary stenosis), the estimated statistical power was 100% and 99.97%, respectively. Removing the 5 cases from the validation tissues dataset that were previously analysed with Illumina EPIC arrays, thus reducing the sample size to 13 BTC and 9 normal tissues, the estimated statistical power remained very high (99.996%).

**Publicly available datasets**

*- TCGA datasets*

Processed Illumina 450K methylation data from The Cancer Genome Atlas (TCGA), including cholangiocarcinoma (TCGA-CHOL, comprising 36 tumour and nine normal controls), colon adenocarcinoma (TCGA-COAD, including 313 tumour and 38 normal controls), rectum adenocarcinoma (TCGA-READ, comprising 98 tumour and seven normal controls) and stomach adenocarcinoma (TCGA-STAD, including 395 tumour and two normal controls) datasets, were downloaded using the Bioconductor package “TCGAbiolinks” (package version: 2.12.6) ^3^. Data were processed following the same pipeline used for the Discovery dataset. Clinical information of TCGA-CHOL cohort is available in Supplementary Table S7.

*- GEO portal dataset*

Processed (Noob background correction and BMIQ normalization) Illumina 450K methylation data from a large BTC study (including 138 tumours and four normal controls) were retrieved from the NCBI Gene Expression Omnibus (GEO) Portal under the accession number GSE89803. Data were downloaded using the Bioconductor package “GEOquery” (package version: 2.52.0) processed following the same pipeline used for Discovery and TCGA-CHOL data. Clinical information of GSE89803 cohort is available in Supplementary Table S8 ^4^.

**Data analyses**

Genome-wide methylation data analysis

Raw DNA methylation data (idat files) were analysed using RnBeads version 2.2.0 ^5^, installed in R environment version 3.6.0, and through *in-house* scripts.

The analysis is divided into several modules: quality control, pre-processing, tracks and tables, exploratory analysis and differential methylation. Background subtraction was performed using the methylumi package (method "enmix.oob") ^6^. Type I and type II scaling was performed using BMIQ method ^7^ implemented in watermelon package version 1.28.0. By default, RnBeads performs the differential methylation analysis with hierarchical linear models as implemented in the limma package ^8^. RnBeads computes *P*-values for all covered CpG sites. The uncorrected CpG-level *P*-values are then combined at the level of predefined genomic regions using a generalization of Fisher’s method ^9^. Aggregate *P*-values are subjected to multiple-testing correction using Bonferroni-Benjamini false discovery rate (FDR).

In addition to the standard output of RnBeads, a custom R script was developed to generate plots to assess the β-values distribution of every sample. A strict filtering was applied to exclude samples 34 samples (33 tumour samples and one normal sample) showing unusual β value distribution (Supplementary Fig. S1).

The final analysis was conducted on high-quality samples, comprising 17 tumour and nine normal tissue samples. CGIs were annotated to the nearest genes and transcripts using R annotation package FDb.InfiniumMethylation.hg19 version 2.2.0 ^10^. Heatmaps were generated by ComplexHeatmap R package version 2.3.4 ^11^. Complete linkage and Euclidean distance were used for clustering. Receiver operating characteristic (ROC) curves were generated by ROCR R package version 1.0.11 ^12^.

Data analysis of ddPCR DNA methylation assay

QuantaSoft software version 1.7.4.0917 (BioRad) was used for ddPCR data analysis. Positive droplet calling was performed using PoDCall algorithm (^13^, available at https://ous-research.no/lind/). The normalized DNA methylation levels were calculated dividing the concentration (copies/µL) of the target gene by the concentration (copies/µL) of the 4Plex control and multiplied by 400. Samples with less than three positive droplets for the target were considered negative for the technical assay. Samples with more than three positive droplets were scored as positive or negative according to a calculated threshold as described below.

ROC curves were generated by IBM SPSS Statistics. The thresholds for scoring samples as methylated were determined based on the respective ROC curves, using the highest sum of sensitivity and specificity are in tissues 2,35 copies/µL for CGI chr2:176993479-176995557 and 1,25 copies/µL for CGI chr5:145713641-145713913 and in bile 0,22 copies/µL for CGI chr2:176993479-176995557.

Sensitivity was calculated as the ratio between tumour positive samples and the total number of tumour samples expressed as percentage. Specificity was calculated as the ratio between non-tumoural negative samples and the total number of non-tumoural samples expressed as percentage.

Best biomarkers identification

We applied a proprietary machine learning approach to select relevant biomarkers as indicators or predictors of disease risk, developing an *in-house* algorithm named TASTOPAL (The Accurate System TO Predict A Lump). Samples were identified as tumour (1) and normal (0) using β values of the selected CGIs as features. This approach combines the markers into an algorithm to accurately estimate the disease risk of an individual, while maintaining a compromise between the constraints given by the experimental and clinical researchers with expertise in DNA methylation and BTC and the best practices suggested by AutoML.

More precisely, TASTOPAL algorithm looks for the best markers under constraints such as the minimum number of markers to be selected to reach the best risk prediction and the application of additional filters explicitly suggested by the researchers. Moreover, it applies automatically several classification algorithms and compares their outputs, in order to find the one performing best on the given datasets. By trying out different learning algorithms on different subsets of markers, and validating results either by cross-validation, or on a different dataset altogether, our program finds the best performing algorithm, identifying in the process the most relevant markers in the resulting classification model. Moreover, the algorithm can process the markers so that the β values are either kept “as-measured” (continuous values), or quantized into a finite set of “step”-values. The experiments have been run with both approaches, i.e. by keeping the continuous values, and by binning them into an on/off binary scale, using an empirically determined threshold of β-value=0.20, with lower values representing unmethylated or “off”, and higher values representing methylated or “on”. Despite binning values into two categories loses some resolution in representing samples, it might result in better generalization and less overfitting, which is especially important if the data have certain level of random noise.

The markers presented in this paper were selected by applying such methodology to the datasets (Discovery dataset, TCGA-CHOL dataset and GSE89803), split as follows: Discovery dataset for training, TCGA-CHOL dataset and GSE89803 for validation. The detailed algorithm is not provided due to copyright restrictions.

The steps carried out by TASTOPAL are synthetized as follow:

| 1  - project ↤ function to use a selection of markers from examples  2  - subset(i, set) ↤ all cardinality i subsets of the given set  3  - CLASSIFIERS ↤ list of classifiers  4  - TRAINING_EXAMPLES ↤ list of examples selected for training  5  - VALIDATION_EXAMPLES ↤ list of examples selected for testing  6  - CANDIDATE_MARKERS ↤ starting list of biomarkers  7  - N ↤ max number of biomarkers to consider  8  - results ↤ empty list  9  - for i in [1..N]  10 -   for markers in subset(i, CANDIDATE_MARKERS)  11 -     best_result ↤ None  12 -     for c in CLASSIFIERS  13 -       for try in [1..M] // try each classifier M times  14 -         model ↤ c.train(project(markers, TRAINING_EXAMPLES))  15 -         c_result ↤ model.test(project(markers,                                             VALIDATION_EXAMPLES))  16 -         if best_result is None or c_result > best_result:  17 -         best_result ↤ c_result  18 -     results.append((markers, best_result))  19 - return results.sorted_by_best_result |
| --- |

Line 3 defines a list of automatic classification algorithms to try, such as logistic regression, kNN, Random Forest, Neural Networks and others.

Line 4 defines the dataset to be used as training set (Discovery dataset), while line 5 defines the validation set (TCGA-CHOL and GSE89803).

Line 7, N is 2 in this case: the query was to obtain 1 or 2 biomarkers. Consequently, line 10 iterates over every subset of 1 or 2 biomarkers. TASTOPAL offers a feature selection mechanism to find the most promising features, without exploring every possible selection. In this case, the number of subsets was small enough to try each set.

In line 12, each classifier is tested several times, trying different hyperparameters.

For each marker, the best result is selected (line 17).

**References**

1. Tsai, P. C., & Bell, J. T. Power and sample size estimation for epigenome-wide association scans to detect differential DNA methylation. *Int. J. Epidemiol.* **44,** 1429–1441 (2015).

2. Fay, M. P. Confidence intervals that match Fisher’s exact or Blaker’s exact tests. *Biostatistics* **11,** 373–374 (2010).

3. Colaprico, A., Silva, T. C., Olsen, C., Garofano, L., Cava, C., Garolini, D., *et al.* TCGAbiolinks: An R/Bioconductor package for integrative analysis of TCGA data. *Nucleic Acids Res.* **44,** e71 (2016).

4. Jusakul, A., Cutcutache, I., Yong, C. H., Lim, J. Q., Huang, M. N., Padmanabhan, N., *et al.* Whole-genome and epigenomic landscapes of etiologically distinct subtypes of cholangiocarcinoma. *Cancer Discov.* **7,** 1116–1135 (2017).

5. Assenov, Y., Müller, F., Lutsik, P., Walter, J., Lengauer, T., & Bock, C. Comprehensive analysis of DNA methylation data with RnBeads. *Nat. Methods* **11,** 1138–1140 (2014).

6. Triche, T. J., Weisenberger, D. J., Van Den Berg, D., Laird, P. W., & Siegmund, K. D. Low-level processing of Illumina Infinium DNA Methylation BeadArrays. *Nucleic Acids Res.* **41,** 1–11 (2013).

7. Teschendorff, A. E., Marabita, F., Lechner, M., Bartlett, T., Tegner, J., Gomez-Cabrero, D., *et al.* A beta-mixture quantile normalization method for correcting probe design bias in Illumina Infinium 450 k DNA methylation data. *Bioinformatics* **29,** 189–196 (2013).

8. Smyth, G. K. Linear models and empirical bayes methods for assessing differential expression in microarray experiments. *Stat. Appl. Genet. Mol. Biol.* **3,** (2004).

9. Makambi, K. H. Weighted inverse chi-square method for correlated significance tests. *J. Appl. Stat.* **30,** 225–234 (2003).

10. Triche, J. T. FDb.InfiniumMethylation.hg19: Annotation package for Illumina Infinium DNA methylation probes. *R Packag. version 2.2.0* (2014).

11. Gu, Z., Eils, R., & Schlesner, M. Complex heatmaps reveal patterns and correlations in multidimensional genomic data. *Bioinformatics* **32,** 2847–2849 (2016).

12. Sing, T., Sander, O., Beerenwinkel, N., & Lengauer, T. ROCR: Visualizing classifier performance in R. *Bioinformatics* **21,** 3940–3941 (2005).

13. Pharo, H. D., Andresen, K., Berg, K. C. G., Lothe, R. A., Jeanmougin, M., & Lind, G. E. A robust internal control for high-precision DNA methylation analyses by droplet digital PCR. *Clin. Epigenetics* **10,** 24 (2018).
